# Remote Ischemic Preconditioning Induces Cardioprotective Autophagy and Signals through the IL-6-Dependent JAK-STAT Pathway

**DOI:** 10.3390/ijms21051692

**Published:** 2020-03-01

**Authors:** Muntasir Billah, Anisyah Ridiandries, Usaid K Allahwala, Harshini Mudaliar, Anthony Dona, Stephen Hunyor, Levon M. Khachigian, Ravinay Bhindi

**Affiliations:** 1 Department of Cardiology, Kolling Institute of Medical Research, Northern Sydney Local Health District, St Leonards, NSW 2065, Australia; anisyah.ridiandries@sydney.edu.au (A.R.); usaid.allahwala@gmail.com (U.K.A.); Harshimudaliar@gmail.com (H.M.); anthony.dona@sydney.edu.au (A.D.); snhunyor@gmail.com (S.H.); ravinay.bhindi@sydney.edu.au (R.B.); 2 Sydney Medical School Northern, University of Sydney, Sydney, NSW 2006, Australia; 3 School of Life Sciences, Independent University Bangladesh, Dhaka 1229, Bangladesh; 4 Vascular Biology and Translational Research, School of Medical Sciences, University of New South Wales, Sydney, NSW 2052, Australia; l.khachigian@unsw.edu.au

**Keywords:** ischemia reperfusion, preconditioning, autophagy, JAK-STAT, interleukin-6

## Abstract

Autophagy is a cellular process by which mammalian cells degrade and assist in recycling damaged organelles and proteins. This study aimed to ascertain the role of autophagy in remote ischemic preconditioning (RIPC)-induced cardioprotection. Sprague Dawley rats were subjected to RIPC at the hindlimb followed by a 30-min transient blockade of the left coronary artery to simulate ischemia reperfusion (I/R) injury. Hindlimb muscle and the heart were excised 24 h post reperfusion. RIPC prior to I/R upregulated autophagy in the rat heart at 24 h post reperfusion. In vitro, autophagy inhibition or stimulation prior to RIPC, respectively, either ameliorated or stimulated the cardioprotective effect, measured as improved cell viability to mimic the preconditioning effect. Recombinant interleukin-6 (IL-6) treatment prior to I/R increased in vitro autophagy in a dose-dependent manner, activating the Janus kinase/signal transducers and activators of transcription (JAK-STAT) pathway without affecting the other kinase pathways, such as p38 mitogen-activated protein kinases (MAPK), and glycogen synthase kinase 3 Beta (GSK-3β) pathways. Prior to I/R, in vitro inhibition of the JAK-STAT pathway reduced autophagy upregulation despite recombinant IL-6 pre-treatment. Autophagy is an essential component of RIPC-induced cardioprotection that may upregulate autophagy through an IL-6/JAK-STAT-dependent mechanism, thus identifying a potentially new therapeutic option for the treatment of ischemic heart disease.

## 1. Introduction

Myocardial infarction (MI) is one of the leading causes of mortality and morbidity worldwide. Timely reperfusion is essential to protect the ischemic myocardium from cell death. Paradoxically, reperfusion can aggravate tissue injury. Brief episodes of non-lethal ischemia to the heart prior to MI can reduce MI damage [1]. This endogenous cardioprotective phenomenon cannot be applied to MI patients, as they present with a blocked coronary artery. Over the last 30 years, thousands of studies on different animal models have identified a vast number of signaling proteins and mediators [2]. In order to improve the outcome of MI patients, it is essential to avoid the damaging effect of I/R injury. From the various alternatives, remote ischemic preconditioning (RIPC) is one of the most promising and attractive non-invasive strategies to attenuate the myocardial damage resulting from ischemia reperfusion (I/R) injury [3]. Accumulating evidence suggests that RIPC strategies can protect not only the human heart but also improve coronary circulation, which has clinical significance [4]. RIPC also demonstrated its cardioprotective potential in high-risk patients with some variability [5,6,7,8,9]. Many studies investigating the impact of RIPC in patients undergoing cardiac surgery showed improved clinical outcomes, such as death, myocardial infarction, stroke, renal failure, length of stay at the intensive care unit, length of mechanical or inotropic support, and length of hospital stay [10]. High-risk patients are most likely to get the largest benefit from RIPC as an adjunctive therapy to percutaneous coronary intervention (PCI) [11]. Daily use of RIPC prevented cardiac remodeling after acute myocardial infarction (AMI) [12], suggesting a prolonged beneficial effect beyond infarct size reduction. A large number of clinical trials have investigated the clinical efficacy of RIPC, with varied results [13]. As an adjunct therapy to standard patient care, RIPC has been demonstrated to reduce cardiac mortality and hospitalization for heart failure patients [14], and improves the myocardial salvage index [15] in ST-elevation myocardial infarction (STEMI) patients. However, a large multi-center trial with cardiac death and hospitalization for heart failure at one year as clinical endpoints in STEMI patients treated with PCI could not deliver any clinical benefit with RIPC [16]. In clinical settings, patients usually have underlying pathological conditions and suffer from multiple comorbidities. These patients are usually treated with multiple medications. As experimental animal models with comorbidity usually does not receive multiple medications for the underlying comorbidity [17], it is difficult to translate the experimental findings to clinical settings. We have previously summarized the effect of RIPC on PCI and coronary artery bypass grafting (CABG) patients [13].

An understanding of the signaling mechanism would be an attractive pharmacological target to be reinforced in situations where cardioprotection is required, such as MI, CABG, and PCI.

Autophagy is a cellular process by which mammalian cells degrade and recycle damaged organelles and proteins [18]. In the heart, autophagy plays a major role in maintaining intracellular homeostasis. Dysregulation of autophagy is associated with several cardiovascular diseases, including ischemic heart disease, cardiac hypertrophy, and heart failure [19]. However, current research suggests that autophagy may be implicated in both the protection and exacerbation of I/R injury following AMI depending on the extent of autophagy induced [20]. A time-dependent upregulation of autophagy in a Langendorff model of rabbit heart subjected to I/R was first described by Decker and Wildenthal in 1980 [21,22]. Inhibition of hypoxia-reoxygenation (H/R)-induced autophagy during reperfusion decreased cardiomyocyte death in vitro [20]. Findings from these previous studies suggested a detrimental effect of autophagy upregulation. However, cardioprotective strategies like ischemic preconditioning and postconditioning have been reported to upregulate autophagy [23,24,25,26]. Perpetually, autophagy inhibition reversed the protective effect of direct ischemic preconditioning and postconditioning [23,26]. Recently, RIPC has also been shown to protect the liver [27], brain [28], and heart [29] from I/R injury by inducing autophagy. However, the pattern of autophagy response varied depending on the extent of the preconditioning stimulus prior to stressing the heart to sustain I/R injury. Previous research has variably linked signal transducer and activator of transcription (STAT)-3 with autophagy regulation [30,31]. We previously demonstrated in a rat model of RIPC that hind limb preconditioning can protect the heart from I/R injury by upregulating the JAK-STAT signaling mechanism [32].

Our group has previously demonstrated that cycles of hypoxia and reoxygenation, which we termed remote hypoxic preconditioning (RHPC), upregulated IL-6 expression and secreted the IL-6 protein into the preconditioned media in vitro [32]. IL-6 is a pleiotropic cytokine stimulated in response to acute injury [33]. Cardiomyocytes express IL-6 [34], and recombinant IL-6 has been demonstrated to be cardioprotective in limiting viral myocarditis in mice [35]. In addition, exercise-mediated upregulation of IL-6 protects against myocardial I/R injury [36]. On the contrary, post-MI and heart failure also chronically elevates IL-6, which is associated with left ventricular (LV) dysfunction and depressed cardiac function [34]. IL-6 has been reported to downregulate starvation-induced autophagy [37], provide chemoresistance in prostate cancer LNCaP cells [38], and protect pancreatic beta cells from apoptosis through autophagy [39]. Moreover, IL-6 activates STAT3 [40], and STAT3-deficient mice showed increased sensitivity to I/R injury [41]. Importantly, preconditioning stimulus confers cardioprotection via IL-6-mediated JAK-STAT pathway activation [42].

The answer of whether RIPC-induced cardioprotection, IL-6, and autophagy upregulation are linked together or are parallel events remains elusive. The current study sought to evaluate the role of autophagy in RIPC, elucidate the underlying mechanisms, and establish the link between circulating IL-6 with cardioprotective autophagy. We found that RIPC prior to I/R upregulates autophagy in vitro and in vivo. RIPC alone also upregulated autophagy in the heart. Inhibition of autophagy prior to H/R failed to confer RIPC-induced cardioprotection, whereas rapamycin-stimulated autophagy mimicked an in vitro preconditioning-like effect. Our study reports the novel findings that RIPC increases circulating IL-6 levels in the blood plasma and regulates cardioprotective autophagy through the myocardial JAK-STAT pathway.

## 2. Results

### 2.1. RIPC Prior to I/R Upregulates Autophagic Activity In Vitro and In Vivo

**Figure 1:**
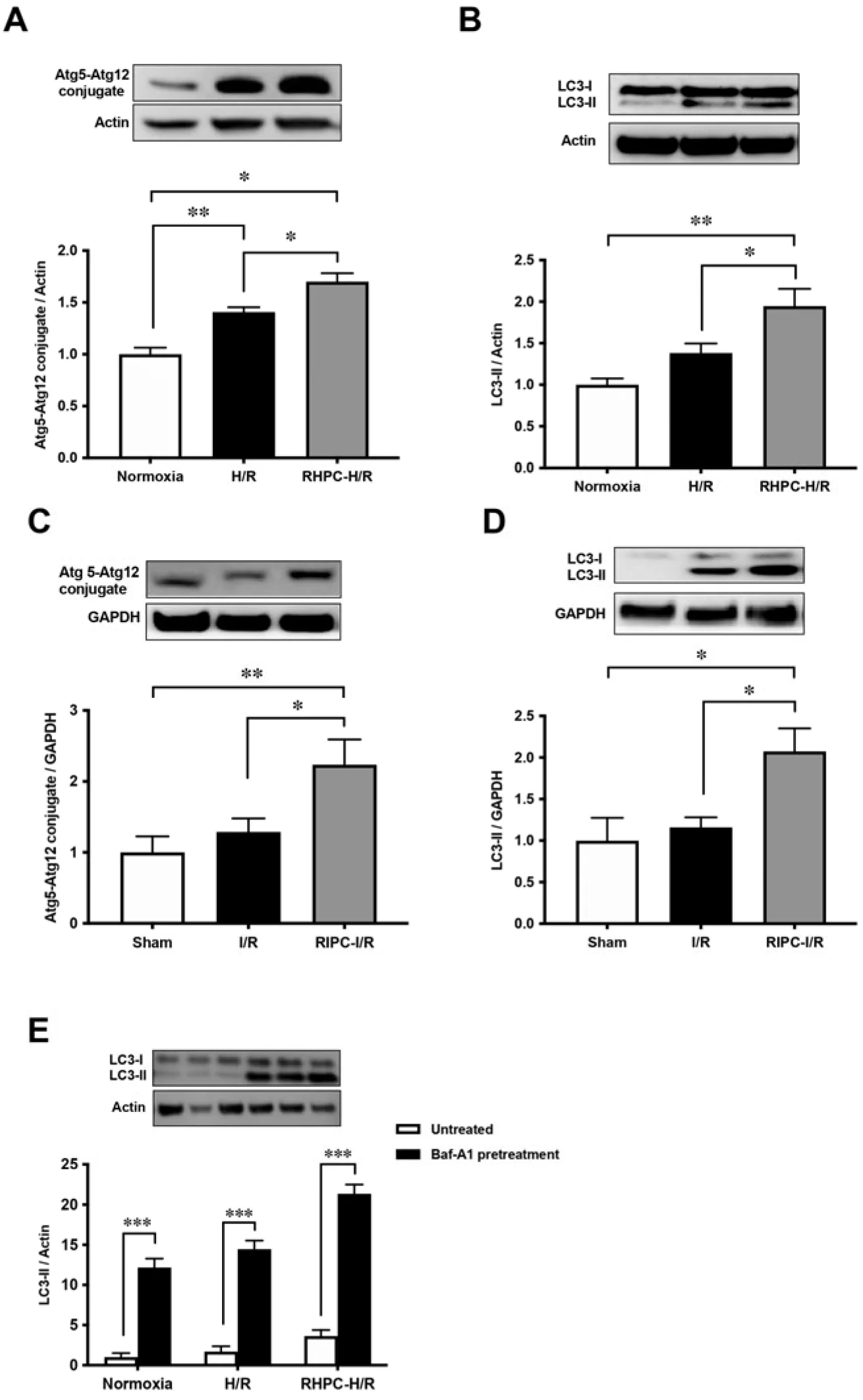
Effect of RIPC prior to I/R on autophagy protein expression in vitro and in vivo. Western blot analysis and representative images of (**A**) Atg5-Atg12 conjugate levels in H9c2 cells, (**B**) LC3 protein levels in H9c2 cells, (**C**) Atg5-Atg12 conjugate levels in rat heart lysate, (**D**) LC3 protein levels in rat heart lysate, and (**E**) LC3 protein levels in bafilomycin-A1-treated H9c2 cells, expressed as mean ± SEM, fold relative to control; *n* = at least 3 independent experiments, * *p* < 0.05, ** *p* < 0.01, *** *p* < 0.001.

To determine autophagic activity, Atg5-Atg12 and LC3-II autophagic markers were assessed in vitro and in vivo. Stimulating with RHPC prior to H/R (RHPC-H/R) in H9c2 cells increased Atg5-Atg12 expression by 1.70 ± 0.08-fold relative to the normoxic control (*p* < 0.05) (Figure 1A). Similarly, LC3-II was significantly increased in RHPC H/R compared to the H/R group (1.95 ± 0.21 vs. 1.38 ± 0.11-fold relative to normoxic control, *p* < 0.05) (Figure 1B). Consistent with the in vitro results, RIPC stimulation in the hindlimb prior to I/R (RIPC I/R) significantly elevated the Atg5-Atg12 conjugate (2.24 ± 0.36 vs. 1.29 ± 0.0.19-fold relative to sham, *p* < 0.05) (Figure 1C) and LC3-II (2.07 ± 0.28 vs. 1.16 ± 0.12-fold relative to sham, *p* < 0.05) (Figure 1D) compared to I/R injury alone. Induction of autophagy was confirmed by pre-treatment of H9c2 cells with bafilomycin A-1 prior to exposing them to H/R. Increased levels of LC3-II in the presence of bafilomycin A-1 are indicative of autophagy flux. However, to assess if H/R and RHPC alter the autophagic flux through substrate digestion, it is important to compare the treatment plus bafilomycin A-1 with the treatment alone group [43]. An additive effect of LC3-II levels with bafilomycin A-1 is suggestive of autophagy flux due to the treatment/intervention; however, if the treatment plus bafilomycin A-1 does not increase LC3-II levels, then it is likely that the autophagy process is impaired [44,45]. In our study, the treatment plus bafilomycin significantly increased LC3-II levels compared to the treatment alone (*p* < 0.001) in all the study groups, suggesting functioning autophagy flux in the normoxia, H/R, and RHPC H/R groups (Figure 1E).

### 2.2. Autophagy Functions as a Signaling Mechanism for RIPC and Confers Cardioprotection Against I/R Injury in Rats

**Figure 2:**
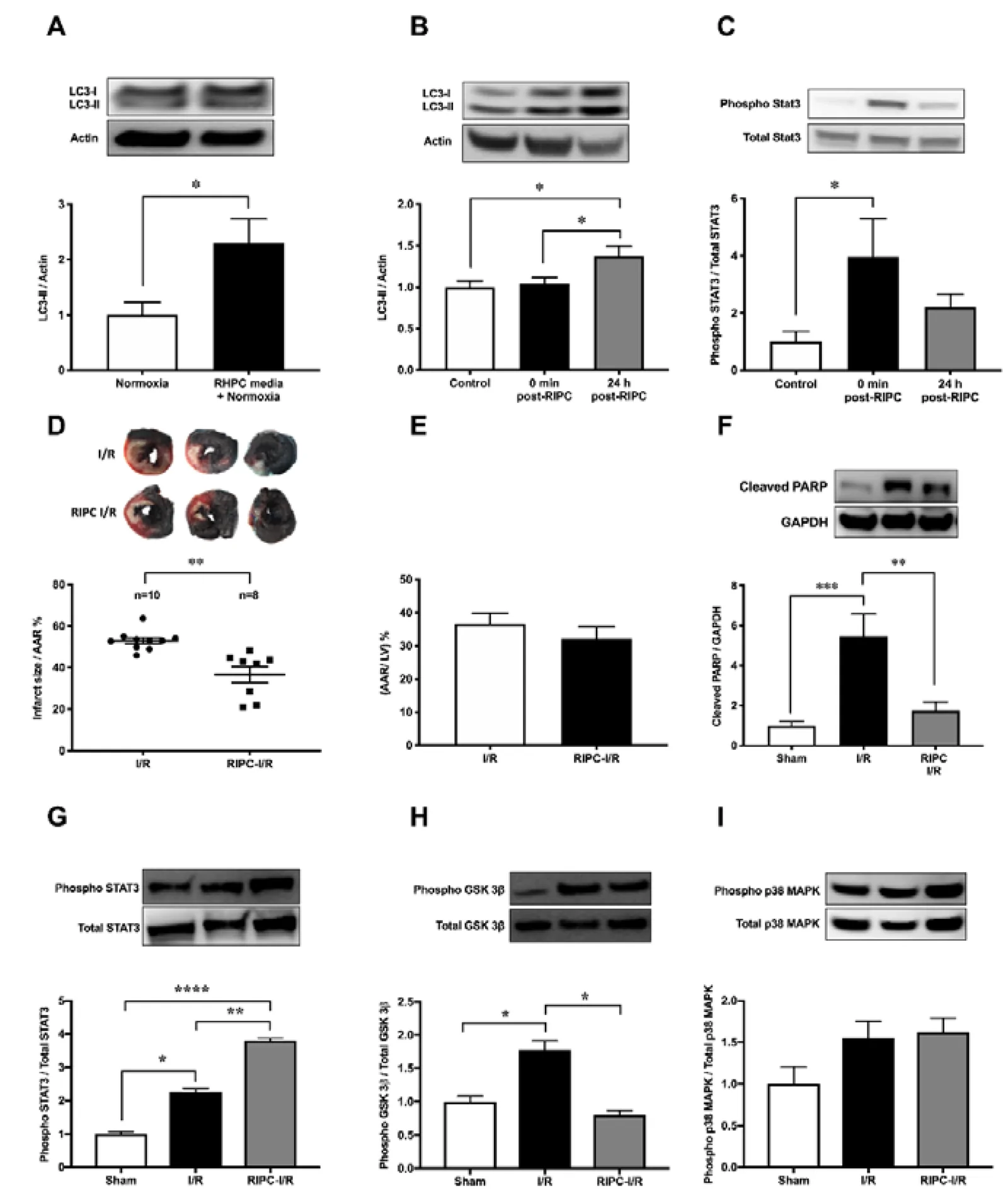
Effect of RIPC on autophagy and the cardioprotective signaling mechanism. Western blot analysis of (**A**) LC3 in H9c2 cells subjected to RHPC (preconditioned) media under normoxic conditions and (**B**) LC3 in the rat heart and (**C**) STAT3 phosphorylation in the rat heart assessed in the control group (without any intervention or sham surgery), at 0 min and 24 h post-RIPC. (**D**–**I**) Rats subjected to I/R with and without prior RIPC. (**D**) MI size expressed as the percentage of infarct size/AAR, (**E**) cumulative data of AAR/LV expressed as the percentage of the mean ± SEM. Western blot analysis of rat heart tissue assessing the (**F**) cleaved PARP (*n* = 8), (**G**) phosphorylated STAT3 (*n* = 8), (**H**) phosphorylated GSK-3β (*n* = 8), and (**I**) phosphorylated p38 MAPK (*n* = 8) levels at 24 h post-I/R with and without prior hindlimb RIPC. Results are expressed as mean ± SEM relative to control; * *p* < 0.05, ** *p* < 0.01, *** *p* < 0.001, **** *p* < 0.0001.

Consistent with the increase of autophagy in H9c2 cells exposed to RHPC-H/R, RHPC alone significantly increased LC3-II protein by 2.29 ± 0.44-fold relative to the normoxic control (*p* < 0.05) (Figure 2A) in vitro. In order to evaluate the contribution of RIPC alone, without left coronary artery (LCA) occlusion and reperfusion, on myocardial autophagy and the cardioprotective JAK-STAT3 pathway, myocardial tissue was assessed for LC3-II and phosphorylated STAT3 levels immediately post-RIPC (0 min post-RIPC) and 24 h post-RIPC. In rats subjected to RIPC only, LC3-II protein in the myocardial tissue increased 1.37 ± 0.13-fold relative to the control group at 24 h post-RIPC (*p* < 0.05 vs. sham, *p* < 0.05 vs. 0 min post-RIPC) (Figure 2B). However, no effect on LC3-II was observed at 0 min post-RIPC compared to the control group (1.04 ± 0.08-fold relative to sham). Interestingly, at 0 min post-RIPC, the autophagy regulator STAT3 was increasingly phosphorylated (3.97 ± 1.33-fold relative to the sham (*p* < 0.05) in myocardial tissue (Figure 2C). However, this value decreased to 2.21 ± 0.45-fold relative to the sham (*p* = 0.32 vs. 0 min post-RIPC) at 24 h post-RIPC.

In confirmation of previous studies, infarct size 24 h following I/R injury was reduced to 36.66 ± 3.87% with RIPC prior to I/R injury compared to I/R injury alone (52.96 ± 1.48%) (*p* < 0.01) (Figure 2D). There was no significant difference in the area at risk (AAR) between groups (Figure 2E). This cardioprotection from RIPC was confirmed by the reduction of cleaved poly(ADP-ribose) polymerase (PARP), an apoptotic marker, in RIPC I/R cardiac tissue compared to I/R alone (1.75 a± 0.44 vs. 5.45 ± 1.12-fold relative to sham, *p* < 0.01) (Figure 2F). Cardioprotective kinase signaling pathways, JAK-STAT, GSK-3β, and p38 MAPK, showed varied outcomes with RIPC prior to I/R. In RIPC I/R, STAT3 phosphorylation was significantly increased compared to I/R only (3.81 ± 0.26 vs. 2.26 ± 0.38-fold relative to sham, *p* < 0.01) (Figure 2G). Interestingly, phosphorylated GSK-3β was significantly elevated in I/R (1.78 ± 0.14-fold relative to the sham group (*p* < 0.05) but reversed to almost the sham group level in RIPC I/R (0.80 ± 0.07-fold relative to the sham group, *p* < 0.05) (Figure 2H). In contrast, p38 MAPK phosphorylation was unchanged after RIPC I/R and I/R alone (Figure 2I).

### 2.3. Autophagy is Essential for RHPC-Induced Cardioprotection In Vitro

**Figure 3:**
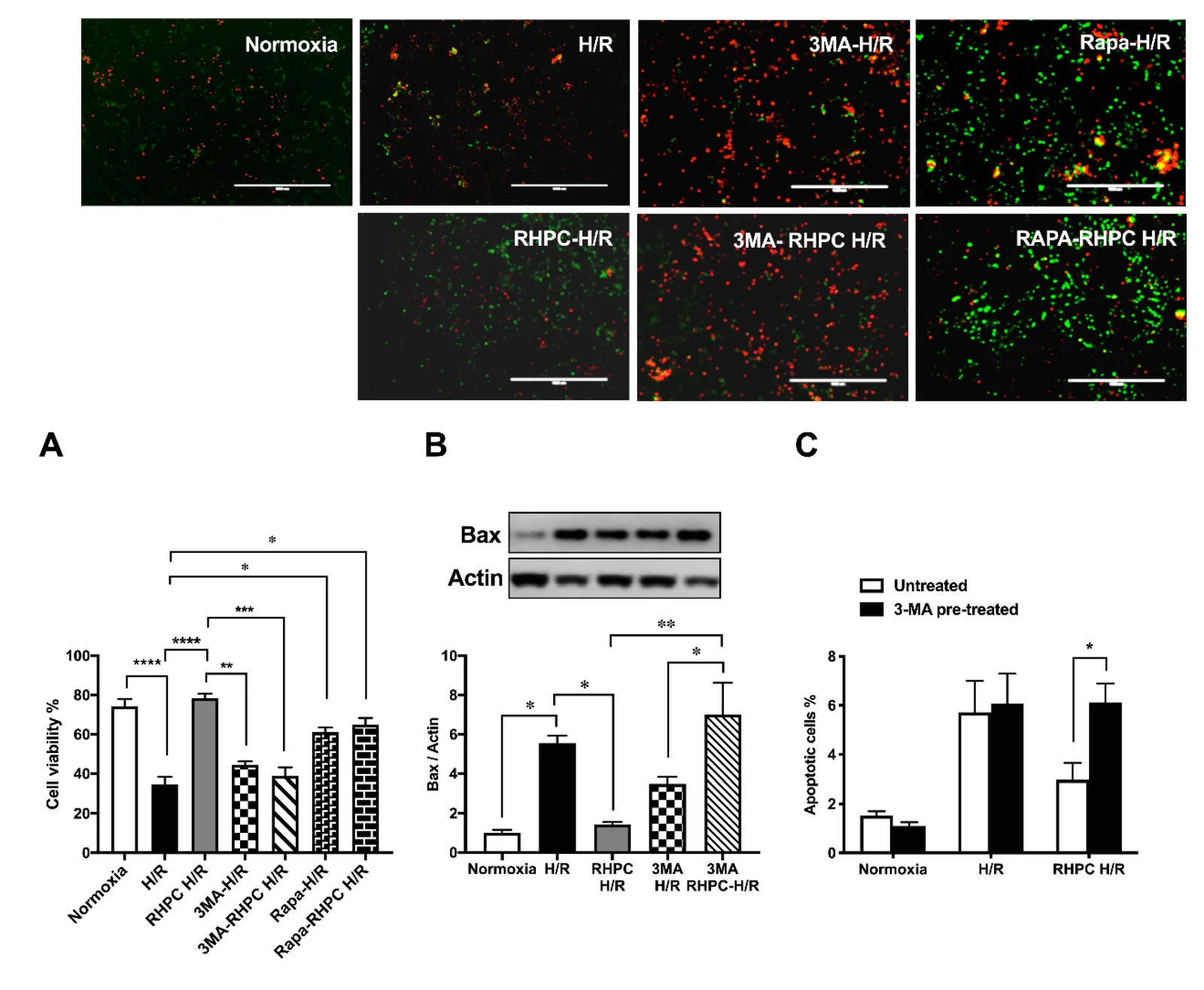

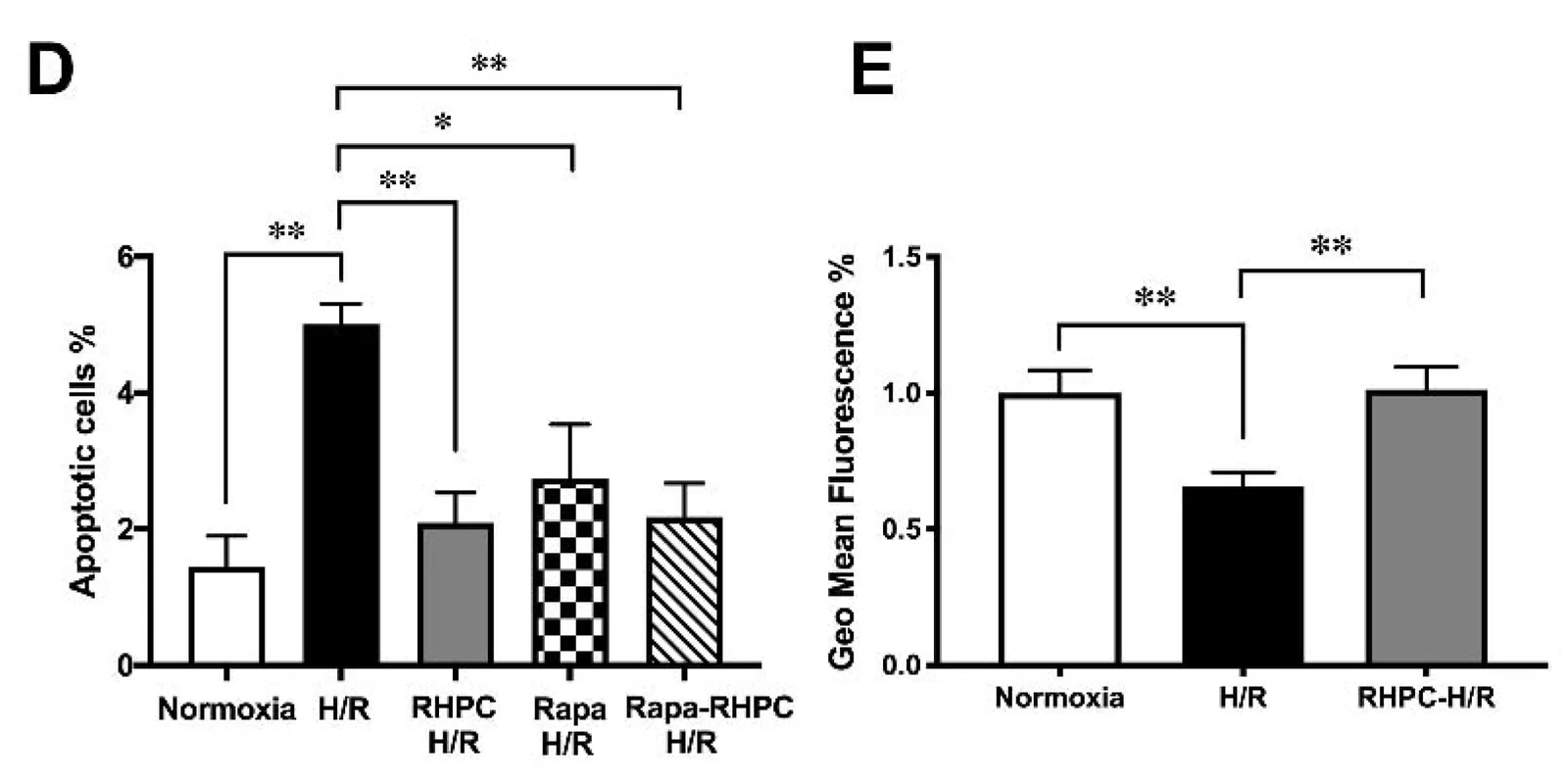
Effect of autophagy modulation on cell viability. (**A**) H9c2 cells were stained with calcein AM (green) and ethidium homodimer-1 (red). Green: live cells, Red: dead cells; the scale bar is 1000 μm. Quantitative analysis of percentage of live/dead cells expressed as mean ± SEM, (**B**) Western blot analysis of pro-apoptotic Bax protein expression are expressed as mean ± SEM, fold relative to the normoxic control, (**C**,**D**) Cell apoptosis assessed by fluorescein isothiocyanate (FITC)-labeled annexin V (annexin V-FITC) and propidium iodide (PI) double staining and fluorescence-activated cell sorting (FACS). Quantitative analysis of percentage of apoptotic cells (annexin-V+/PI+) cells are represented as mean ± SEM; (**E**) Mitochondrial membrane potential at 1 h post-reoxygenation assessed by 1,1′,3,3,3′,3′-hexamethylindodicarbocyanine iodide (DiLC5) staining and FACS represented as the percentage geo mean fluorescence ± SEM, fold relative to the normoxia control; *n* = at least 3 independent experiments, * *p* < 0.05, ** *p* < 0.01, *** *p* < 0.001, **** *p* < 0.0001.

To assess the importance of autophagy in the RIPC protective effect, H9c2 cells were pre-treated with 3-Methyladenine (3-MA) (inhibits autophagy) or rapamycin (Rapa) (promotes autophagy) prior to stimulation in the H/R injury model (Figure 3A). Pre-treatment with 3-MA significantly reduced cell viability in 3-MA RHPC-H/R cells compared to RHPC-H/R alone (38.89 ± 4.44% vs. 78.43 ± 3.68%, *p* < 0.0001). In contrast, pre-treating the cells with rapamycin significantly reduced cell viability in the RAPA-RHPC-H/R group compared to RHPC-H/R alone (64.93 ± 3.47% vs. 78.43 ± 3.68%, *p* < 0.05) and in Rapa H/R compared to H/R (61.32 ± 2.22 vs. 34.83 ±3.63, *p* < 0.05). Consistent with these results, pro-apoptotic Bax protein expression was significantly increased with 3-MA RHPC-H/R treatment compared to RHPC-H/R alone (7.02 ± 1.60 vs. 1.44 ± 0.11-fold relative to the normoxic control, *p* < 0.01) (Figure 3B). Additionally, 3-MA pre-treatment resulted in a significant increase in H9c2 apoptosis in the RHPC-H/R group compared to the untreated RHPC-H/R group (6.13 ± 0.77% vs. 2.97 ± 0.69%, *p* < 0.05) (Figure 3C). However, treatment with rapamycin prior to H/R resulted in a significant decrease in apoptosis in the H/R group compared to the untreated H/R group (2.75 ± 0.79% vs. 5.01 ± 0.30%, *p* < 0.05) (Figure 3D). Changes in the mitochondrial membrane potential triggers the cycle of reactive oxygen species (ROS) formation [46], and is linked with cell death [47,48]. We analyzed the mitochondrial membrane potential level in H9c2 cells with and without RHPC prior to H/R. The mitochondrial membrane potential was lost in the H/R group (0.66 ± 0.05%-fold relative to control, *p* < 0.01); however, the mitochondrial membrane potential was maintained in the RHPC-H/R group (1.01 ± 0.08%-fold relative to control, *p* < 0.05 vs. H/R) (Figure 3E).

### 2.4. RIPC-Induced Autophagy Regulated by the IL-6-Dependent JAK-STAT Pathway

**Figure 4:**
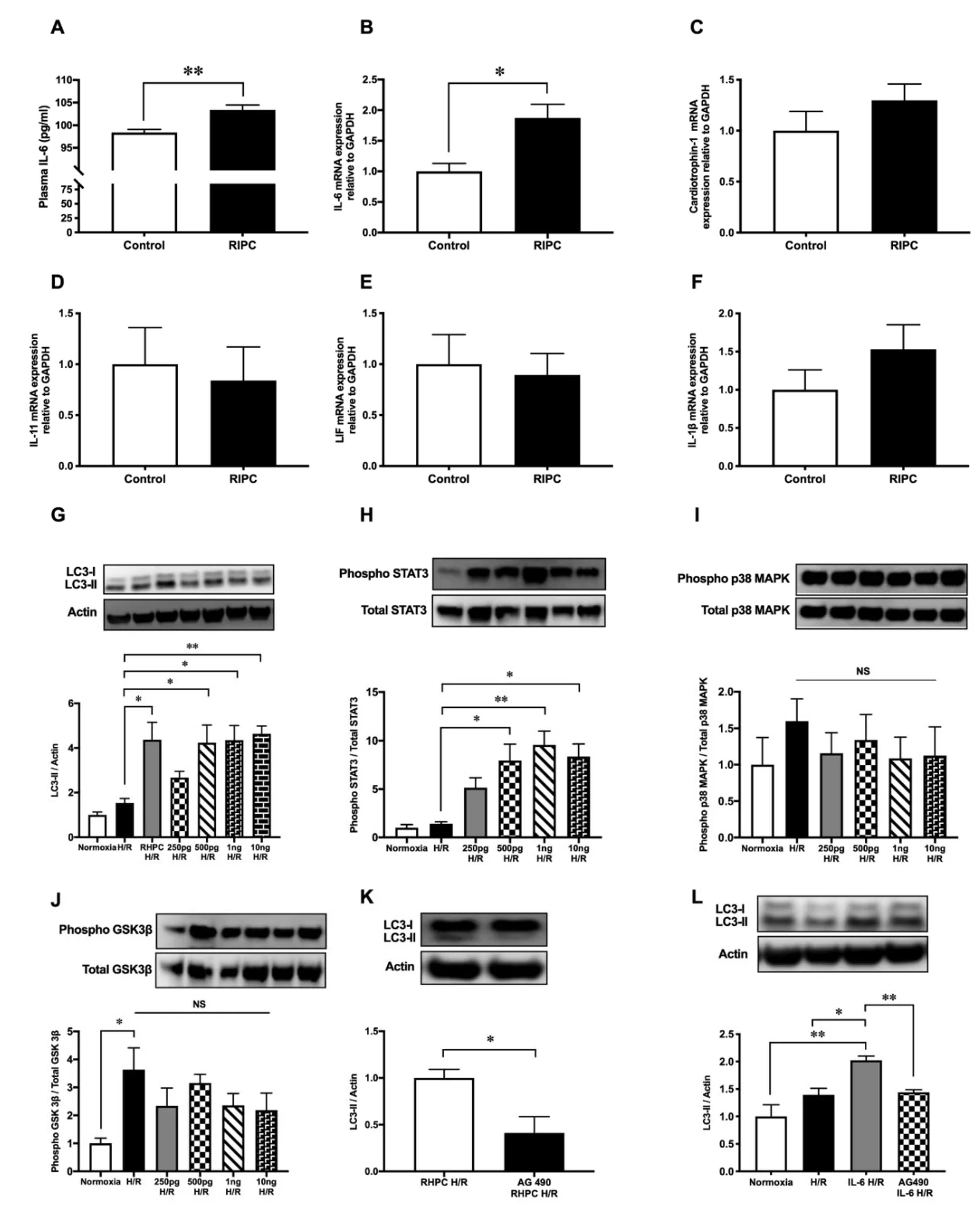
RIPC modulates cytokines’ expression and recombinant IL-6 treatment induces autophagy through the JAK-STAT pathway. (**A**) Plasma IL-6 protein levels at 0 min post-RIPC expressed as mean ± SEM, *n* = 5, *p* < 0.01. mRNA expression of (**B**) IL-6, (**C**) cardiotrophin-1, (**D**) IL-11, (**E**) LIF, and (**F**) IL-1β in rat hind limb muscle at 0 min post-RIPC assessed by qPCR and expressed as mean ± SEM; *n* = 9, * *p* < 0.05. Western blot analysis of (**G**) LC3, (**H**) phosphorylated STAT3, (**I**) phosphorylated GSK-3β, and (**J**) phosphorylated p38 MAPK in H9c2 cells pre-treated with different concentrations of IL-6 (250 pg/mL, 500 pg/mL, 1 ng/mL, 10 ng/mL) during hypoxia followed by reoxygenation. The expression levels of phosphorylated proteins were normalized to total STAT3, GSK-3β, and p38 MAPK, respectively. Results are expressed as mean ± SEM; * *p* < 0.05, ** *p* < 0.01. H9c2 cells exposed to RHPC-H/R with and without the JAK-STAT pathway inhibitor tyrphostin AG-490. Representative immunoblots and statistical data of (**K**) LC3 protein are expressed as mean ± SEM, fold relative to the RHPC-H/R group. (**L**) Representative immunoblot of the LC3 protein level in H9c2 cells with IL-6 treatment during hypoxia with and without tyrphostin AG-490 pre-treatment followed by reoxygenation are expressed as mean ± SEM, fold relative to the normoxic control; *n* = at least 3 independent experiments, * *p* < 0.05, ** *p* < 0.01.

After RIPC, the secretion of cytokines critical in cardiovascular pathology was assessed. In rats subjected to RIPC, circulating IL-6 protein levels in the plasma were significantly increased to 103.4 ± 1.08 pg/mL compared to 98.4 ± 0.68 pg/mL in the sham group (*p* < 0.01) (Figure 4A). Similarly, in rat hindlimb tissue where RIPC was performed, localized messenger RNA (mRNA) IL-6 expression was upregulated by 1.87 ± 0.22-fold relative to the sham group (*p* < 0.05, Figure 4B). In contrast, RIPC did not modulate the expression of other critical cytokines in cardiovascular pathology, including cardiotrophin-1 (Card-1) (1.30 ± 0.16-fold relative to the sham group) (Figure 4C), interleukin-11 (IL-11) (0.84 ± 0.33-fold relative to the sham group) (Figure 4D), leukemia inhibitory factor (LIF) (0.89 ± 0.21-fold relative to the sham group) (Figure 4E), and IL-1β (1.53 ± 0.32-fold relative to the sham group) (Figure 4F).

When H9c2 cells were treated with increasing doses of recombinant IL-6, LC3-II protein was upregulated compared to the untreated H/R group (*p* < 0.05) (Figure 4G). However, the LC3-II protein levels in the IL-6-treated H/R groups were similar to the RHPC-H/R group. Similarly, increasing concentrations of IL-6 treatment during hypoxia followed by reoxygenation increased STAT3 phosphorylation in a dose-dependent manner (Figure 4H). However, recombinant IL-6 treatment during hypoxia followed by reoxygenation did not modulate the phosphorylation level of p38 MAPK (Figure 4I) and GSK-3β (Figure 4J).

Pre-treatment of H9c2 cells with the STAT3 inhibitor tyrphostin AG-490 (50μM) prior to RHPC-H/R significantly reduced the RHPC-H/R-induced increase in LC3-II protein levels compared to the untreated RHPC-H/R group (0.41 ± 0.17-fold relative to the untreated RHPC-H/R group, *p* < 0.05) (Figure 4K). Tyrphostin AG-490 pre-treatment prior to H/R failed to upregulate LC3-II protein levels despite pre-treating H9c2 cells with 500 pg/mL of recombinant IL-6 during the hypoxic period prior to exposure to reoxygenation (1.44 ± 0.05 vs. 2.02 ± 0.08-fold relative to the normoxic control, *p* < 0.01) (Figure 4L).

## 3. Discussion

In this study, we highlight the importance of autophagy in the initiation of RIPC prior to I/R injury. Our in vivo studies confirm that autophagy is associated with reduced infarct size in RIPC. Additionally, RIPC increases autophagy at the myocardium. We found that RIPC induces the expression of IL-6 at the preconditioned hindlimb muscle and increases plasma IL-6 secretion in vivo, a finding that aligns with our previous in vitro findings [32].

Serum provides an optimum growing environment for cells [49,50,51,52]. However, the composition of serum is complex, and contains various known and unknown factors that may confound the experimental findings. It is possible to minimize the analytical interference from unknown variables by omitting or growing the cells in serum-free conditions prior to exposing the cells to the experimental condition. Serum starvation also provides more reproducible experimental conditions [53,54]. In addition, serum-free conditions apparently reduce basal cellular activity [55], and the growing cell population becomes more homogenous and synchronized by entering the quiescent G_0_/G_1_ phase of the cell cycle [56,57]. In the current study, overnight serum starvation was used as a preparatory measure. During the experiments, serum- and glucose-free medium was used to mimic the nutrition deficiency aspect of AMI [58,59]. In our study, we serum starved the cells overnight by lowering the serum concentration in Dulbecco’s modified Eagle’s medium (DMEM) from 10% to 1%. Other research groups also followed a similar technique to serum starve H9c2 cells overnight prior to exposing them to H/R injury [32,60]. In order to minimize any bias or potential confounding effect from overnight serum starvation, we serum starved all the experimental and control groups, and maintained a homogenous condition across the groups prior to the experiments. Nonetheless, complete serum starvation in H9c2 cells can inhibit apoptosis, and promote cell proliferation and cell cycle progression through Rac1 protein [61]. However, in a particular study by Zhao and colleagues, they completely serum starved (0% fetal bovine serum (FBS)) H9c2 cells for 48 h, which does not align with our experimental condition.

Stimulators, such as oxidative stress, intracellular Ca^2+^ overload, rapid restoration of physiological pH at the time of reperfusion, and mitochondrial permeability transition pore (mPTP) pore opening, all operate in the first few minutes of myocardial reperfusion, providing a narrow window for reducing myocardial MI size. However, ischemia-induced apoptosis and inflammation continue over several hours into reperfusion and may contribute to lethal myocardial reperfusion injury. Myocardial reperfusion injury is a progressive injury, and experimental data demonstrated an increase in MI size with increasing reperfusion time well beyond coronary occlusion and reflow [62,63]. Myocardial reperfusion injury is a dynamic injury with peak myeloperoxidase (MPO) activity and endothelial dysfunction at 24 h [64]. RIPC has two windows of protection: A first or early window of protection opens within minutes of RIPC stimulus and remains open for 4–5 h, whereas the second window of protection opens at a later time point after RIPC stimulus and remains open for some days [13]. Autophagy was regarded as being involved in non-apoptotic programmed cell death and was considered a doubled-edged sword in cell survival [65,66]. Previous studies suggested autophagy as being cardioprotective in myocardial ischemic injury; however, autophagy during reperfusion causes cell death [67]. Our group has previously published studies on rat myocardial I/R injury with 24 h of reperfusion [32]. Hence, assessing reperfusion injury at 24 h allows the wide therapeutic window of protection to mitigate the dynamic and progressive reperfusion injury. We observed a non-significant increase in autophagy in the heart after I/R injury at 24 h (Figure 1C,D). We showed that RIPC prior to I/R further upregulated autophagy in the heart tissue and was associated with a smaller infarct size (Figure 2D). Consistent with our findings, chloramphenicol succinate conferred cardioprotection against I/R injury by upregulating autophagy in a swine model [68]. Furthermore, autophagy is prevalent in viable tissue of the chronically ischemic myocardium [69]. However, recent reports suggest that RIPC prior to CABG failed to activate autophagy in LV myocardium despite activated cardioprotective signaling cascades [70].

To further determine the importance of autophagy induction in RHPC, rapamycin was used to promote while 3-MA was used to inhibit autophagy prior to RHPC-H/R in vitro [71,72]. This study demonstrated that rapamycin treatment significantly reduced apoptosis and improved cell viability in both the H/R and RHPC-H/R groups, suggesting that autophagy upregulation can mimic the cardioprotective effect against H/R injury (Figure 3A,D). In contrast, 3-MA pre-treatment almost completely suppressed the protective effect of RHPC as suggested by the significant increase in apoptosis and decline in cell viability in the RHPC-H/R group (Figure 3A–C). However, 3-MA pre-treatment did not have any significant effect on the H/R group, suggesting that autophagy might not have any contributing role in the deleterious effect of H/R injury in this study.

We found that RIPC alone increased autophagy in the rat heart at 24 h (Figure 2B). Although, Gedik and colleagues reported that autophagy is not involved in RIPC-induced protection in patients undergoing CABG surgery [70]. However, Gedik’s group only studied autophagy at baseline before initializing CABG and at 5–10 min post-aortic reperfusion, hence they did not take early or late activation of autophagy into consideration. In our model, we did not observe any significant change in autophagy levels immediately post-RIPC; however, at 24 h post-RIPC, we observed a significant upregulation in autophagy activity. In our study, JAK-STAT pathway activation immediately after RIPC (Figure 2C) suggests that cardioprotective kinase pathways’ activation precedes autophagy machinery activation, and RIPC-induced autophagy may have a signaling role in cardioprotection. A recent study in mice by Ghani and colleagues reported that RIPC induces cardiac stress and accumulates cardiac adenosine prior to cardiac ischemia [73], which further supports our findings.

We found that RIPC induces the expression of IL-6 at the preconditioned hindlimb muscle and increases plasma IL-6 secretion in vivo (Figure 4A,B), a finding that aligns with our previous in vitro findings [32]. However, Gedik and colleagues did not detect any significant change in IL-6 levels in arterial plasma sample collected after RIPC from patients undergoing elective CABG [74]. Age, presence of co-morbidity, medications, anesthetic regimen, and extent of preconditioning stimuli may all interfere with the cardioprotective modalities of RIPC [13] and may explain the discrepancy with our findings. In addition, the timing of sampling may have interfered with the detection of IL-6 protein after RIPC stimuli. We found a 5 pg/mL difference of RIPC-induced plasma IL-6 was correlated with cardioprotection and myocardial autophagy in vivo. However, in vitro, the 250 pg/mL recombinant IL-6 treatment did not have any significant effect on autophagy. The apparent differences are likely due to the different experimental conditions in vitro vs. in vivo. In response to ischemia, the endogenous STAT3 level increases; however, the ischemia-induced increase in STAT3 is not sufficient to protect the mitochondria [75]. Nonetheless, overexpression of STAT3 has been shown to protect the mitochondria during ischemia [75]. We demonstrated that RIPC alone can increase STAT3 phosphorylation in the heart beyond that of I/R injury (Figure 2C). We also found that RHPC prior to H/R maintained the mitochondrial membrane potential in vitro (Figure 3E)*,* suggesting preserved mitochondrial function. Recent studies have documented the role of STAT3 in autophagy regulation [31]. To substantiate this link, increasing doses of IL-6 were found to increase phosphorylation of STAT3 (Figure 4H), whereas inhibition of the JAK-STAT pathway with tyrphostin AG-490 failed to upregulate autophagy despite pre-treatment with IL-6 during the hypoxic period (Figure 4L). It is likely that, RIPC mediates autophagy through an IL-6/JAK-STAT-dependent axis, and different protocols and procedures of RIPC-induced cardioprotection may use a distinctive pattern of STAT isoform signaling and humoral mediators.

We also investigated the p38 MAPK (Figure 4I) and GSK3β (Figure 4J) signaling pathways, which have been implicated in myocardial I/R injury and RIPC [76,77,78]. We aimed to delineate if the p38 MAPK and GSK3β pathways have any role in regulating RIPC-induced autophagy. Our results suggest no link between increased p38 MAPK phosphorylation and an increase in LC3-II protein levels in RIPC-I/R. This contrasts with previous studies that have implicated p38 MAPK phosphorylation in the induction of autophagy [79]. GSK-3β is activated during the ischemic phase by dephosphorylation at serine-9 (Ser-9) whereas it is deactivated by phosphorylation at Ser-9 during reperfusion. Ser-9 phosphorylation negatively regulates the activity of GSK-3β [80]. Activated GSK-3β is known to stimulate autophagy during ischemia whereas inactivation of GSK-3β inhibits autophagy during reperfusion [81], suggesting that the dephosphorylated state of GSK-3β has a cardioprotective role during the ischemic phase through upregulation of autophagy. In contrast to the findings of Hu and colleagues, who demonstrated that increased phosphorylation of GSK-3β at Ser-9 (increased inactivity) post-RIPC is associated with cardioprotection [82], we observed a significant decrease in phosphorylated GSK-3β (increased activity) in the RIPC I/R group. Increasing doses of IL-6 did not demonstrate any significant change in the phosphorylation level of both p38 MAPK and GSK-3β, suggesting no involvement of the p38 MAPK and GSK-3β pathways in IL-6-mediated autophagy regulation.

Activation of autophagy is associated with an increase in LC3-II and decrease in p62 protein [43]. In addition, p62 acts as a platform for LC3-positive structures via binding to LC3-I proteins in the cells [83], making both LC3 and p62 valuable markers of autophagy [84]. However, we did not observe any Western blot signal for p62 protein in our study. As we did not observe any impaired autophagy in our study (Figure 1E), it is possible that increased autophagy by RIPC degraded the p62 protein below the level of detection. Future studies may use autophagy and lysosomal inhibitors and assess the level of p62 protein, which may provide a more accurate scenario of p62 protein levels in cells.

Anesthesia is a confounder of cardioprotection by RIPC [85]. A meta-analysis by Zangrillo and colleagues reported that volatile anesthesia combined with RIPC reduced the post-operative mortality rate in patients undergoing cardiac surgery [86]. However, a meta-analysis by Zhou and colleagues reported an attenuation of RIPC-induced protection in cardiac surgery patients exposed to volatile anesthesia. [87]. Volatile anesthetics, such as isoflurane, sevoflurane, and propofol, have been previously reported to precondition the myocardium and also upregulate autophagy [88,89]. Isoflurane preconditioning relies on an acute memory phase, and discontinuation of isoflurane 30 min prior to left anterior descending (LAD) artery occlusion resulted in cardioprotection [90]. Isoflurane exposure before, but not during, prolonged LAD artery reduced the myocardial infarction size similar to ischemic preconditioning. However, whether the residual isoflurane remains in the myocardial tissue after 30 min of its discontinuation is debatable. Volatile anesthesia had no effect on the myocardial infarct size when applied throughout the procedure (continuous anesthesia) compared to intermittent anesthesia, which reduced infarct size [91,92,93]. Sheng and colleagues reported that 3 h of isoflurane exposure increased autophagy in mouse cortical neurons [94]. However, Li and colleagues reported that isoflurane exposure for 1 h did not have any effect on hippocampal autophagy levels in aged rats. In this study, we applied continuous isoflurane (2% *v/v*) for three cycles of 5 min intermittent hind limb ischemia and reperfusion, and during the 30 min of LCA occlusion, totaling around 1 h of isoflurane exposure, which may not be long enough to implicate any direct effect on the autophagy levels observed in our study. In addition, we exposed all the animal groups (including the sham surgery group) to continuous isoflurane to eliminate any possibility of inter-animal variation unrelated to the treatment group. However, future studies with a similar anesthetic protocol are essential to assess the impact of isoflurane exposure on infarct size reduction.

The translation of cardioprotection to the clinical setting is a challenging issue. Heusch and colleagues reported STAT3 activation in [95] pigs but not in [96] humans. Previous studies demonstrated that STAT5 but not STAT3 activation is associated with the signaling mechanism of RIPC in humans, suggesting the presence of species-specific differences in the signaling mechanism [96]. Pepe and colleagues performed RIPC in children as young as 1 month (average 7 months age) undergoing tetralogy of Fallot repair, and found no differences in phosphorylated Akt or STAT3 between the control and RIPC groups [97]. Yet, Wu and colleagues demonstrated significant STAT3 activation in similar clinical settings but with older children (average 10- to 11-month-old children) [98]. Long-term regular RIPC was demonstrated to increase STAT3 in arterial samples discarded from patients undergoing CABG [99].

## 4. Study Limitations

This study did not modulate autophagy in vivo to analyze its effect on RIPC-induced cardioprotection. Further experiments are needed to establish the therapeutic window of cardioprotection by autophagy activation to clinically translate autophagy to therapeutic settings. Though acute ischemic injury also causes myocardial injury [100], this study focused on the effect of autophagy regulation on RIPC-induced protection from reperfusion injury, hence studying the effect of autophagy regulation on ischemic injury was beyond the scope of this paper. IL-6/JAK-STAT pathway involvement in the activation of autophagy in vitro was demonstrated in this study. Partial evidence was provided for in vivo as well. However, replicating the total in vitro findings in vivo using knock-out mice is a possibility for a future study. RIPC has previously been demonstrated to improve coronary vasodilation in pigs [101], microcirculation in both healthy volunteers and patients with heart failures [102], and LV function and remodeling in patients at risk of large myocardial infarcts [11]. However, an assessment of cardiac function pre- and post-RIPC in our animal model was beyond the scope of the current study and warrants future studies. Currently, techniques to monitor autophagy flux in vivo are still underdeveloped and the available techniques cannot reliably replicate the in vitro findings in the in vivo setting. It is possible to assess autophagy in vivo by using GFP-LC3 transgenic animals or by transfecting with GFP-LC3 plasmids [103,104]. However, this technique requires tissue specificity and caution is warranted to interpret the autophagy level in the heart when the specified animal models are used. We did not inhibit IL-6 prior to RHPC to assess the effect on protection and autophagy. We did, however, use different concentrations of recombinant IL-6 protein prior to H/R and demonstrated that it mimics a similar effect as RHPC-induced protection and RHPC-induced autophagy. Further studies that explore the levels of STAT3 and autophagy following RIPC in the presence of IL-6 antibody are necessary to consolidate the proposed mechanism of IL-6/STAT3-dependent RIPC-induced autophagy. The current study is a proof of concept of RIPC-mediated cardioprotection through IL-6-dependent autophagy. While we did not assess the combined effect of RHPC and recombinant IL-6 on autophagy, it is possible that both have such effects, and are more pronounced with RIPC, in which case other factors may contribute to cardioprotection. However, assessing the combined effect of RHPC and recombinant IL-6 on the autophagy level was beyond the scope of the current study. In addition, we did not specifically study the effect of RIPC on selective degradation of mitochondria by autophagy (mitophagy), which warrants future investigation. In order to replicate the in vivo findings in a controlled in vitro environment, we used H9c2 cells in our study. H9c2 cells are widely used to study H/R injury [105] and are more closely connected to cardiomyocytes than HL-1 cells in terms of their energy metabolism [106]. H9c2 cells have biochemical and electrophysiological properties of both cardiac and skeletal tissues [107], hence they have previously been used as an in vitro model for both skeletal and cardiac tissue [108]. There are challenges in using an in vitro cell line as a model with respect to the translation of mechanistic insight. Embryonic or neonatal cardiomyocytes and undifferentiated myoblast cell lines, such as H9c2 cells, can undergo apoptosis; however, it is unclear whether studying apoptosis and relevant targets in H9c2 cells can be relevant to cardioprotective interventions in humans [109]. Though the in vivo rat hind limb was preconditioned to induce a cardioprotective effect, in our study, we preconditioned H9c2 cells in media. We have previously published the use of H9c2 cells as an in vitro RHPC model [32]. In vivo RIPC stimulus involves neuronal, humoral, and systemic mediators to induce cardioprotection [13], while our in vitro model relies on the transfer of soluble mediators of preconditioning, which does not completely mimic the in vivo RIPC signal transfer mechanism. Besides apparent static vs. flow differences, the in vivo setting is three-dimensional and includes various cell types and a range of soluble mediators, unlike the in vitro setting, which although it is more controlled, it has regimented change by way of exposure of one cell type to one cytokine and new media. We used this simplistic in vitro approach to study the signaling mechanism; however, this limitation can be overcome in future studies by incorporating other myocardial cells, including fibroblasts, endothelial cells, and pericytes to cardiomyocytes, in a co-culture model.

The proteasome, a highly sophisticated protease complex, degrades unneeded and damaged proteins. The ubiquitin proteasome system (UPS) and autophagy are two major intracellular degradation pathways. Though it was generally assumed that UPS and autophagy serve distinct functions, major investigations have revealed shared mechanisms and interplay between these two proteolytic systems in the heart [110]. UPS not only degrades damaged proteins but also regulates various cellular processes [111]. UPS becomes dysfunctional as a result of I/R injury [112]; however, ischemic preconditioning preserves the UPS function [113]. However, we did not assess changes in the proteasome pathway, and it was beyond the scope of the current study. Future studies may explore the contribution of UPS to RIPC-induced cardioprotection.

## 5. Materials and Methods

In order to ensure the rigor and reproducibility in preclinical and clinical studies on cardioprotection, practical guidelines published by Botker and colleagues were followed for the mitochondrial membrane potential analysis and in vivo I/R model [109].

### 5.1. In Vitro H/R Injury Model

RHPC and H/R were performed as previously described [32,114]. ‘Hypoxic media’ was directly bubbled with nitrogen for 15 min to achieve less than 2% O_2_ followed by tightly closing the lid. Normoxic media was not bubbled with nitrogen. To stimulate RHPC, H9c2 cells grown to 70–80% confluency were exposed to 3 × 5 min alternating cycles of hypoxia (<2% O_2_) and reoxygenation. During hypoxia, hypoxic media was added to H/R cells and exposed to 30 min of hypoxia in the hypoxic chamber. The chamber was flushed with nitrogen to maintain the hypoxic environment inside the chamber. Restoration of oxygenation (reoxygenation) exacerbates hypoxic injury [115]. Reoxygenation but not hypoxia alone is a strong apoptotic stimuli [116]. In accordance with the previously published model of 30-min hypoxia and 1-h reoxygenation of cardiomyocytes [32,117], we exposed the overnight serum-starved H9c2 cells to 30 min of hypoxia. After 30 min of hypoxia, cells were washed with warm Dulbecco’s Phosphate-Buffered Saline (D-PBS), and exposed to normoxic media for 1 h in normoxic conditions (20% O_2_, 37 °C). The RHPC-H/R group received preconditioned media during the 30-min hypoxia followed by 1-h reoxygenation in normoxic media. The normoxia group received the normoxic media for 1.5 h at 37 °C. RHPC and H/R injury was achieved in hypoxic (HEPES-buffered DMEM, pH 7.4 with no glucose) and normoxic buffer (HEPES-buffered DMEM, pH 6.5 with 10mM glucose).

### 5.2. In Vivo I/R Injury Model

**Figure 5:**
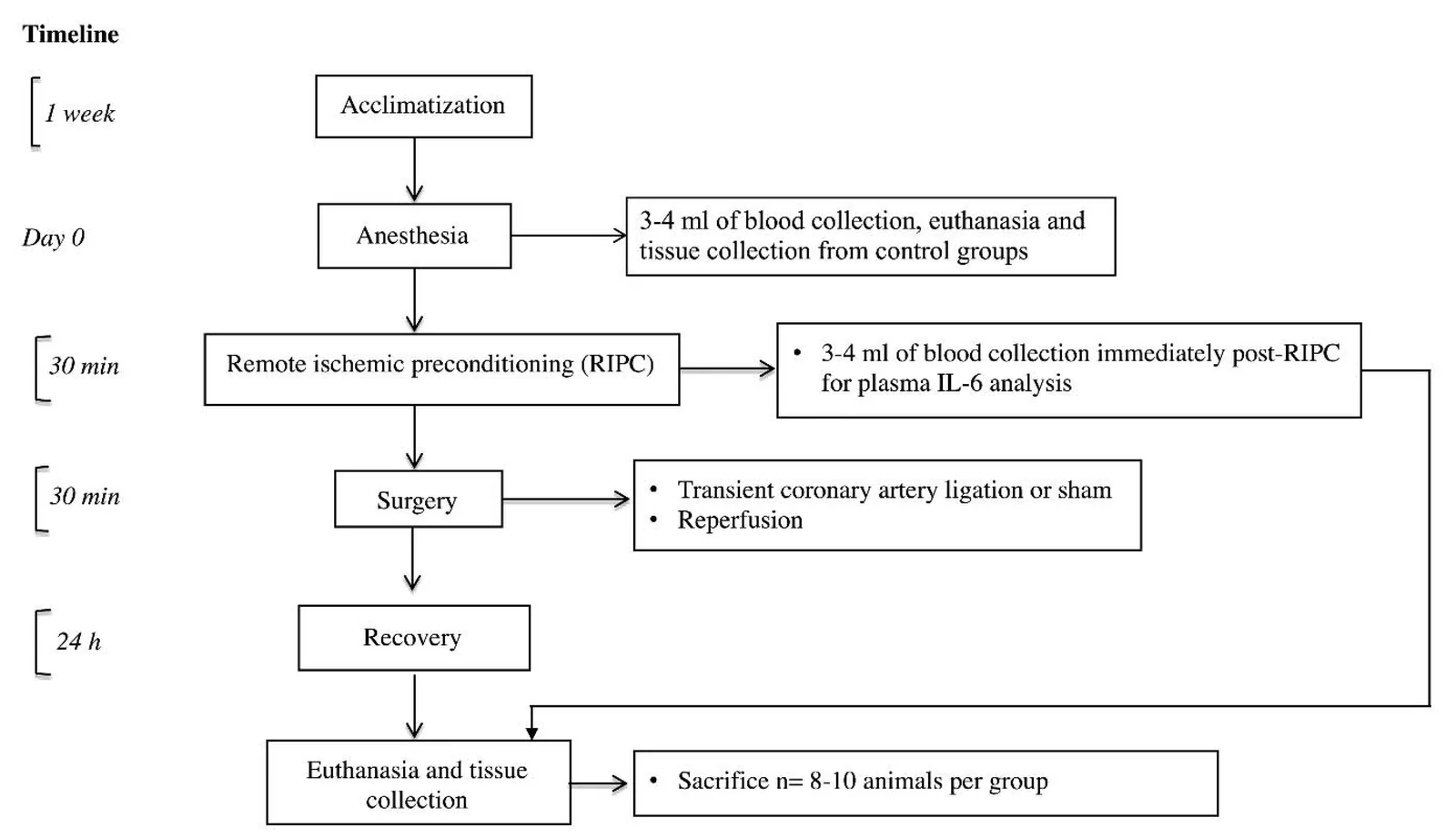
Schematic timeline of the in vivo experiments.

Experiments in this study were approved by the Animal Care and Ethics Committee of Royal North Shore Hospital on 20th February 2014 (Approval code 1401-004A). Experiments were performed according to the recommendations of the Australian Council for Animal Care. A schematic timeline of the in vivo experiments is shown in Figure 5.

Briefly, male Sprague Dawley rats (300–350 g) were separated into 3 groups: Sham, I/R injury, and RIPC-I/R. In this study, we used isoflurane as it provides rapid induction and safe recovery from anesthesia with comparatively negligible effects on cardiovascular parameters and respiratory rates [118]. Isoflurane anesthesia was induced and maintained as previously recommended by Guo and colleagues [119]. Anesthesia was induced in an induction chamber using 5% (*v/v*) vaporized isoflurane. The animal was positioned supine, and endotracheal intubation performed using an 18-gauge plastic cannula. Anesthesia was maintained with 2% (*v/v*) isoflurane and the animal ventilated at a rate of 80 breaths per min with a tidal volume of 1.5 mL per 100 g of body weight using a small animal ventilator. Immediately prior to surgery, an intramuscular injection of lignocaine (10 mg per kg body weight) and subcutaneous injection of Temgesic (0.1 mg per kg body weight) was administered. Animals in the RIPC group were subjected to RIPC prior to I/R injury by fastening a tourniquet around the hindlimb for 3 × 5 min alternating cycles of ischemia (tightening tourniquet) and reperfusion (loosening tourniquet), where cyanosis and a drop in the temperature of the foot confirmed limb ischemia. To induce I/R injury, a thoracotomy was performed at the left 5th intercostal space to expose the heart. After removal of the pericardium, the LCA was ligated using a 6/0 silk suture to induce 30 min of ischemia as confirmed by myocardial cyanosis. The suture was then released to induce reperfusion. The chest cavity was closed, and the animal monitored for 24 h. Subcutaneous injection of temgesic (0.1 mg per kg body weight) was administered every 8 h till euthanasia. For the sham group, the surgical technique was identical, but the LCA was not ligated. For the control group, no sham surgery was performed. The AAR was determined post-experiment with Evan’s blue and tetrazolium chloride (TTC).

### 5.3. Measurement of Myocardial Infarct Size and Tissue Collection

Rats were anesthetized and intubated. Sutures were removed, and self-retaining retractor was used to re-open the chest cavity. LCA was re-occluded and 5mL of 3% Evans blue in D-PBS solution was injected into the tail vein, and allowed to perfuse for 3 to 5 min. The heart was excised, and washed in D-PBS. The excised heart was wrapped in food cling film, with the heart placed in an Eppendorf vial and placed in a container, and kept on dry ice for no more than 10 min, which made the heart semi-frozen [120]. Frozen or semi-frozen hearts have been previously used by other research groups to measure infarct sizes in both rats and mice [120,121,122,123,124,125]. Food wrap prevents the heart from freeze-drying. Freeze-dried tissue is likely to appear as tetrazolium chloride (TTC) negative. However, freeze-dried tissue can easily be recognized by the presence of an unstained epicardial layer in all the slices and in both ischemic and non-ischemic regions. Semi-frozen heart was sliced into 2-mm-thick slices using a rat heart slicer matrix. In our study, we did not observe any freeze-drying effect on the heart slices. Slicing the semi-frozen heart bypassed the laborious process of embedding the heart in agarose to facilitate slicing [126]. Freezing the heart at −20 °C for up to 2h has no effect on the intensity of TTC staining [120]. TTC perfusion causes severe tissue contracture, which may prevent successful dye perfusion. In order to minimize this, we immersed the heart sections in freshly prepared 1% TTC in D-PBS solution for 10 min each side at 37 °C followed by immersion in 10% neutral formal buffer to increase the contrast, which were examined and digitally photographed as previously described [120]. Immersion in neutral formalin buffer enhances the red/pale contrast between the viable AAR and area of necrosis, and removes the fatty surface gloss [120]. Images were analyzed using ImageJ. Non-AAR appeared as blue. Area that did not appear as blue was considered as AAR. TTC stained viable myocardium as red/pink and infarcted tissue appeared as white. We measured the infarct size as the percentage of infarct size/AAR. In order to make sure that the AAR was equal in the infarcted animals, we measured the ratio of AAR over the LV size and observed no significant difference between the I/R and RIPC I/R groups. A linear relationship exists between the body weight and the heart weight of rats at 10 weeks of age [127]. All our animals were between 300–350 g of weight and aged 8–11 weeks. We used a rat heart slicer matrix and sliced the heart into 2-mm-thick slices. However, the weight of each slice was not calculated. As we used specific weighted and aged rats, and cut the heart in equal size slices, it is expected that the variance in terms of the weight of slices across the groups was minimal. In addition, the percentage of infarct size/AAR has been widely used to measure infarct size in vivo [32,114,128,129]. Hind limb tissue at the RIPC site was collected and immediately frozen in dry ice.

### 5.4. Assessment of Autophagy

We interpreted the autophagy data according to the guidelines published by Klionsky and colleagues [43]. During autophagy, a cytosolic form of LC3 (LC3-I) is conjugated with phosphatidylethanolamine (PE) to form autophagosome-bound LC3-II. LC3-II is located in both the exterior and lumen of the autophagosome. Luminal LC3-II is degraded by the fusion of the autophagosome with lysosomes, while LC3-II on the cytoplasmic surface can be delipidated and recycled. LC3-II is only associated with autophagosomes and not with any other vesicular structures. Therefore, LC3-II levels correlate with the autophagic vacuole numbers [130]. Though assessment of early and late autophagic vacuoles by immunocytochemistry would generate valuable data regarding the overall status of autophagy in the cells, it was beyond the scope of the current study. We rather studied the level of autophagosome formation and autophagy flux in response to RIPC and rat myocardial I/R injury.

### 5.5. Western Blotting

Protein expression of Atg5, LC3-II, cleaved PARP, Bax, STAT3, GSK-3β, and p38 MAPK in apical heart tissue and H9c2 cell lysate were measured using western immunoblot. Separate animals were used to extract protein from the myocardial apical tissue and to measure infarct size. Cells were washed with cold D-PBS and trypsinized. Trypsinized cells were centrifuged at 1,000 rpm for 5 min at 4ºC and supernatant discarded. Cell pellet was resuspended in radioimmunoprecipitation assay (RIPA) cell lysis buffer containing 50mM TRIS hydrochloride (Tris-HCL), 150mM Sodium chloride (NaCl), 5mM ethylenediaminetetraacetic acid (EDTA) (pH 7.4), 0.5% Triton X-100, and protease and phosphatase inhibitor (Roche Diagnostics, Manheim, Germany). For tissue lysate, extracted tissue was homogenized in RIPA buffer containing protease and phosphate inhibitor. Cell/tissue lysate was centrifuged at 14,000 rpm for 15 min at 4 °C and supernatant stored at −80 °C. Protein quantification was carried out using a bicinchoninic acid (BCA) protein estimation kit (Thermofisher Scientific, Waltham, MA, USA) in accordance with the manufacturer’s instruction. Protein was prepared for electrophoresis by mixing protein sample with 10× Dithiothreitol (DTT) (Life technologies, Carlsbad, CA, United States) and 4× gel loading buffer (Life technologies) and boiling samples at 70 °C for 5 min. An equal amount of protein was resolved using Bolt Bis-Tris gel for 1 h at 150V. Depending on the separation required, either 2-(N-morpholino)ethanesulfonic acid (MES) or 3-(N-morpholino)propanesulfonic acid (MOPS) buffer was used as running buffer. Proteins were transferred to Hybond nitrocellulose membranes (Amersham Pharmacis Biotech, Bucks, UK) using Bolt transfer buffer (Life technologies) by the wet transfer method. Transfer membranes were first stained with Ponceau S to confirm successful protein transfer and even loading, and then the membranes were cut into smaller sections [131]. The smaller sections of the membrane were blocked in Tris-buffered saline with 0.5% Tween-20 (TBST) in either 5% skim milk or 5% BSA (for phospho-proteins) for 1 h and then incubated overnight at 4 °C with the primary antibodies. Primary and secondary antibodies were diluted in TBST. Membranes were washed 3 × 10 min with TBST and incubated with 1:5,000 horseradish peroxidase-conjugated secondary antibody in either 5% skim milk or 5% BSA (for phosphorylated proteins) for 2 h at room temperature. Membranes were washed 3 × 10 min with TBST. Proteins were visualized using a Supersignal West Pico Chemiluminescent substrate (ECL) kit (Thermofisher Scientific, Waltham, MA, USA) in an LAS 4000 machine. Semi-quantitative analysis was performed using Multigauge software (Fujifilm, Minato City, Tokyo, Japan). Even protein loading was confirmed by probing for either β-actin for cell lysate or GAPDH for heart tissue lysate except the phosphorylated proteins. For the phosphorylated proteins, we used the total expression of the same protein to accurately determine the equivalent protein loading. A protein’s phosphorylation status may change through modification of the protein’s phosphorylation level [132] or changes in the total amount of the protein present [133]. If the treatment affects both the phosphorylated and total protein level, then the ratio of phosphorylated to total protein remains constant. Hence, normalization of the phosphorylated proteins to the total expression of the same protein allows a more reliable and accurate assessment of the phosphorylated status of the protein. Total proteins have been widely used to confirm even loading [134,135]. We have previously published using the total expression of protein to demonstrate equal loading for phosphorylated proteins without any issue [32,114].

### 5.6. Flow Cytometry

In H9c2 cells subjected to an in vitro H/R model, the level of cellular apoptosis was assessed using a FITC Annexin V Apoptosis Detection Kit (556547, BD Biosciences, Franklin Lakes, NJ, USA) model. To assess the mitochondrial membrane potential loss, H9c2 cells were collected and then suspended in D-PBS, 5μL of 2μM stock of DiLC5 dye was added to the cells, and cells were incubated at room temperature for 15 min in the dark. The fluorescence intensity of the cells was analyzed by using a BD FACS flow cytometer.

### 5.7. Cell Viability

Cells from the H/R in vitro model were pre-treated with 3-MA, rapamycin, or no pre-treatment, and assessed for cell viability using the Live/Dead Cell Imaging kit (R37601; Thermofisher Scientific, Waltham, MA, USA). Fluorescence images were taken using green fluorescent protein (GFP) and Texas Red filter in an EVOS FL Auto Cell Imaging System (Thermofisher Scientific, Waltham, MA, USA).

### 5.8. Real-Time Polymerase Chain Reaction

Hindlimb tissue was homogenized in Trizol reagent (15596026; Thermofisher Scientific, Waltham, MA, USA) using TissueRuptor (Qiagen, Hilden, Germany) and RNA was extracted as per the manufacturer’s instruction. Quantitative RT-PCR was performed in triplicate on an ABI Prism 7900 HT Sequence Detection System (Applied Biosystems, Foster City, CA, USA). The average expression of the gene of interest from three replicate extractions was expressed relative to the housekeeping gene. Table 1 includes the primer sequences of the genes used for RT-PCR in this study. 

### 5.9. Enzyme-Linked Immunosorbent Assay

A rat IL-6 Quantikine ELISA Kit (R6000B; R&D Systems, Minneapolis, MN, USA) was used to measure plasma IL-6 in rat blood collected immediately post-RIPC.

### 5.10. Autophagy Promotion and Inhibition

To promote autophagy, serum-starved H9c2 cells were treated with 1μM rapamycin (R0395; Sigma Aldrich, St. Louis, MO, USA) (Supplementary Figure S1) for 4 h, whilst to inhibit autophagy serum-starved H9c2 cells were treated with 10 nM 3-MA (M9281; Sigma Aldrich) for 2 h (Supplementary Figure S2). Lysosomal digestion of the autophagosomes, through fusion of the lysosomes with autophagosomes and degradation of the contents, is a key step of the autophagy mechanism, and assessing the completion of this step is a crucial part of evaluating complete autophagy (autophagy flux) [43]. To assess autophagy flux, cells were treated with 10 nM Bafilomycin A1 (Baf-A1), a lysosomal inhibitor, for 4 h. To evaluate the effect of IL-6 on autophagy, H9c2 cells were exposed to hypoxic media containing recombinant IL-6 during 30 min of hypoxia. Additionally, H9c2 cells were treated with 200μM tyrphostin AG-490 (T3434; Sigma Aldrich) (Supplementary Figure S3) for 1 h prior to exposing the cells to H/R to inhibit the JAK-STAT pathway as previously described [32].

### 5.11. Statistical Analysis

Normalized results are expressed as mean ± standard error of the mean (SEM). We initially assessed the normality of the data distribution. If the data were normally distributed, then statistical analysis between groups was performed by one-way ANOVA, followed by post hoc comparison using Tukey’s multiple comparison tests using GraphPad Prism software (GraphPad software version 7.02; GraphPad Inc, San Diego, CA, USA). If the data were not normally distributed, we performed a Kruskal–Wallis test followed by Dunn’s multiple comparisons test. Comparison between two groups was performed using the two-tailed Student’s t-test. *p* ≤ 0.05 was considered to be significant.

## 6. Conclusions

Modulation of autophagy can be a novel, intriguing, and potential powerful molecular strategy to reduce I/R injury. Collectively, the findings of our study support an essential role for autophagy in RIPC-mediated cardioprotection. Autophagy may also mediate heart resistance to cellular stress by activating cardioprotective signaling mechanisms. In addition, our study proposes IL-6 as a possible mediator of autophagy in RIPC and suggests a possible mechanism for the induction of autophagy.

## Figures and Tables

**Table 1:** Primer sequences used for RT-PCR.

Gene	Primer Sequence	Annealing Temperature
Cardiotrophin-1	Forward 5′-AGCCTTCCTGATTTCTCAGGCT-3′ Reverse 5′-AATT TCTGTGTTGGCGCAGTGTGG-3′	60 °C
LIF	Forward 5′-TGTGGTAGCAGCCGAGTCATAAT-3′ Reverse 5′-CGTGGCTTGTGTGTTCGGTTTCAT-3′	60 °C
IL-6	Forward 5′-GCTCGTCGTCGACAACGGCCTC-3′ Reverse 5′-CAAACATGATCTGGGTCATCTTCTC-3′	60 °C
IL-11	Forward 5′-CGTGAAGCTGTGTTGTCCTG-3′ Reverse 5′-GCTCCTAGGACTGTCTTCTTC-3′	60 °C
IL-1β	Forward 5′-CTGTGACTCGTGGGATGATG-3′ Reverse 5′-GGGATTTTGTCGTTGCTTGT-3′	60 °C
GAPDH	Forward 5′-ATGGGAAGCTGGTCATCAAC-3′ Reverse 5′ GTGGTTCACACCCATCACAA-3′	60 °C

## Supplementary Materials

Supplementary materials can be found at https://www.mdpi.com/1422-0067/21/5/1692/s1.

## References

[1] Murry C.E., Jennings R.B., Reimer K.A. (1986). Preconditioning with ischemia: A delay of lethal cell injury in ischemic myocardium. Circulation.

[2] Heusch G. (2015). Molecular basis of cardioprotection: Signal transduction in ischemic pre-, post-, and remote conditioning. Circ. Res..

[3] Heusch G. (2017). Critical Issues for the Translation of Cardioprotection. Circ. Res..

[4] Kleinbongard P., Skyschally A., Heusch G. (2017). Cardioprotection by remote ischemic conditioning and its signal transduction. Pflug. Arch..

[5] Ahmad A.M., Ali G.S., Tariq W. (2014). Remote ischemic preconditioning is a safe adjuvant technique to myocardial protection but adds no clinical benefit after on-pump coronary artery bypass grafting. Heart Surg Forum.

[6] Karuppasamy P., Chaubey S., Dew T., Musto R., Sherwood R., Desai J., John L., Shah A.M., Marber M.S., Kunst G. (2011). Remote intermittent ischemia before coronary artery bypass graft surgery: A strategy to reduce injury and inflammation?. Basic Res. Cardiol..

[7] Hong D.M., Lee E.H., Kim H.J., Min J.J., Chin J.H., Choi D.K., Bahk J.H., Sim J.Y., Choi I.C., Jeon Y. (2014). Does remote ischaemic preconditioning with postconditioning improve clinical outcomes of patients undergoing cardiac surgery? Remote Ischaemic Preconditioning with Postconditioning Outcome Trial. Eur. Heart J..

[8] Lucchinetti E., Bestmann L., Feng J., Freidank H., Clanachan A.S., Finegan B.A., Zaugg M. (2012). Remote ischemic preconditioning applied during isoflurane inhalation provides no benefit to the myocardium of patients undergoing on-pump coronary artery bypass graft surgery: Lack of synergy or evidence of antagonism in cardioprotection?. Anesthesiology.

[9] McCrindle B.W., Clarizia N.A., Khaikin S., Holtby H.M., Manlhiot C., Schwartz S.M., Caldarone C.A., Coles J.G., Van Arsdell G.S., Scherer S.W. (2014). Remote ischemic preconditioning in children undergoing cardiac surgery with cardiopulmonary bypass: A single-center double-blinded randomized trial. J. Am. Heart Assoc..

[10] Botker H.E., Lassen T.R., Jespersen N.R. (2018). Clinical translation of myocardial conditioning. Am. J. Physiol. Heart Circ. Physiol..

[11] Munk K., Andersen N.H., Schmidt M.R., Nielsen S.S., Terkelsen C.J., Sloth E., Botker H.E., Nielsen T.T., Poulsen S.H. (2010). Remote Ischemic Conditioning in Patients with Myocardial Infarction Treated with Primary Angioplasty: Impact on Left Ventricular Function Assessed by Comprehensive Echocardiography and Gated Single-Photon Emission CT. Circ. Cardiovasc. Imaging.

[12] Wei M., Xin P., Li S., Tao J., Li Y., Li J., Liu M., Li J., Zhu W., Redington A.N. (2011). Repeated remote ischemic postconditioning protects against adverse left ventricular remodeling and improves survival in a rat model of myocardial infarction. Circ. Res..

[13] Billah M., Ridiandries A., Allahwala U., Mudaliar H., Dona A., Hunyor S., Khachigian L.M., Bhindi R. (2019). Circulating mediators of remote ischemic preconditioning: Search for the missing link between non-lethal ischemia and cardioprotection. Oncotarget.

[14] Gaspar A., Lourenco A.P., Pereira M.A., Azevedo P., Roncon-Albuquerque R., Marques J., Leite-Moreira A.F. (2018). Randomized controlled trial of remote ischaemic conditioning in ST-elevation myocardial infarction as adjuvant to primary angioplasty (RIC-STEMI). Basic Res. Cardiol..

[15] Botker H.E., Kharbanda R., Schmidt M.R., Bottcher M., Kaltoft A.K., Terkelsen C.J., Munk K., Andersen N.H., Hansen T.M., Trautner S. (2010). Remote ischaemic conditioning before hospital admission, as a complement to angioplasty, and effect on myocardial salvage in patients with acute myocardial infarction: A randomised trial. Lancet.

[16] Hausenloy D.J., Kharbanda R.K., Moller U.K., Ramlall M., Aaroe J., Butler R., Bulluck H., Clayton T., Dana A., Dodd M. (2019). Effect of remote ischaemic conditioning on clinical outcomes in patients with acute myocardial infarction (CONDI-2/ERIC-PPCI): A single-blind randomised controlled trial. Lancet.

[17] Kleinbongard P., Botker H.E., Ovize M., Hausenloy D.J., Heusch G. (2019). Co-morbidities and co-medications as confounders of cardioprotection—Does it matter in the clinical setting?. Br. J. Pharmacol..

[18] Mizushima N., Levine B., Cuervo A.M., Klionsky D.J. (2008). Autophagy fights disease through cellular self-digestion. Nature.

[19] Xie M., Morales C.R., Lavandero S., Hill J.A. (2011). Tuning flux: Autophagy as a target of heart disease therapy. Curr. Opin. Cardiol..

[20] Valentim L., Laurence K.M., Townsend P.A., Carroll C.J., Soond S., Scarabelli T.M., Knight R.A., Latchman D.S., Stephanou A. (2006). Urocortin inhibits Beclin1-mediated autophagic cell death in cardiac myocytes exposed to ischaemia/reperfusion injury. J. Mol. Cell Cardiol..

[21] Decker R.S., Poole A.R., Crie J.S., Dingle J.T., Wildenthal K. (1980). Lysosomal alterations in hypoxic and reoxygenated hearts. II. Immunohistochemical and biochemical changes in cathepsin D. Am. J. Pathol..

[22] Decker R.S., Wildenthal K. (1980). Lysosomal alterations in hypoxic and reoxygenated hearts. I. Ultrastructural and cytochemical changes. Am. J. Pathol..

[23] Gurusamy N., Lekli I., Gorbunov N.V., Gherghiceanu M., Popescu L.M., Das D.K. (2009). Cardioprotection by adaptation to ischaemia augments autophagy in association with BAG-1 protein. J. Cell. Mol. Med..

[24] Huang C., Yitzhaki S., Perry C.N., Liu W., Giricz Z., Mentzer R.M., Gottlieb R.A. (2010). Autophagy induced by ischemic preconditioning is essential for cardioprotection. J. Cardiovasc. Transl. Res..

[25] Huang C., Andres A.M., Ratliff E.P., Hernandez G., Lee P., Gottlieb R.A. (2011). Preconditioning involves selective mitophagy mediated by Parkin and p62/SQSTM1. PLoS ONE.

[26] Wagner C., Tillack D., Simonis G., Strasser R.H., Weinbrenner C. (2010). Ischemic post-conditioning reduces infarct size of the in vivo rat heart: Role of PI3-K, mTOR, GSK-3beta, and apoptosis. Mol. Cell. Biochem..

[27] Wang Y., Shen J., Xiong X., Xu Y., Zhang H., Huang C., Tian Y., Jiao C., Wang X., Li X. (2014). Remote ischemic preconditioning protects against liver ischemia-reperfusion injury via heme oxygenase-1-induced autophagy. PLoS ONE.

[28] Su J., Zhang T., Wang K., Zhu T., Li X. (2014). Autophagy activation contributes to the neuroprotection of remote ischemic perconditioning against focal cerebral ischemia in rats. Neurochem. Res..

[29] Rohailla S., Clarizia N., Sourour M., Sourour W., Gelber N., Wei C., Li J., Redington A.N. (2014). Acute, delayed and chronic remote ischemic conditioning is associated with downregulation of mTOR and enhanced autophagy signaling. PLoS ONE.

[30] You L., Wang Z., Li H., Shou J., Jing Z., Xie J., Sui X., Pan H., Han W. (2015). The role of STAT3 in autophagy. Autophagy.

[31] Jonchere B., Belanger A., Guette C., Barre B., Coqueret O.C. (2013). STAT3 as a new autophagy regulator. Jak-Stat.

[32] Mudaliar H., Rayner B., Billah M., Kapoor N., Lay W., Dona A., Bhindi R. (2017). Remote ischemic preconditioning attenuates EGR-1 expression following myocardial ischemia reperfusion injury through activation of the JAK-STAT pathway. Int. J. Cardiol..

[33] Tanaka T., Narazaki M., Kishimoto T. (2014). IL-6 in inflammation, immunity, and disease. Cold Spring Harb. Perspect. Biol..

[34] Fontes J.A., Rose N.R., Cihakova D. (2015). The varying faces of IL-6: From cardiac protection to cardiac failure. Cytokine.

[35] Kanda T., McManus J.E., Nagai R., Imai S., Suzuki T., Yang D., McManus B.M., Kobayashi I. (1996). Modification of viral myocarditis in mice by interleukin-6. Circ. Res..

[36] McGinnis G.R., Ballmann C., Peters B., Nanayakkara G., Roberts M., Amin R., Quindry J.C. (2015). Interleukin-6 mediates exercise preconditioning against myocardial ischemia reperfusion injury. Am. J. Physiol. Heart Circ. Physiol..

[37] Qin B., Zhou Z., He J., Yan C., Ding S. (2015). IL-6 Inhibits Starvation-induced Autophagy via the STAT3/Bcl-2 Signaling Pathway. Sci. Rep..

[38] Chang P.C., Wang T.Y., Chang Y.T., Chu C.Y., Lee C.L., Hsu H.W., Zhou T.A., Wu Z., Kim R.H., Desai S.J. (2014). Autophagy pathway is required for IL-6 induced neuroendocrine differentiation and chemoresistance of prostate cancer LNCaP cells. PLoS ONE.

[39] Linnemann A.K., Blumer J., Marasco M.R., Battiola T.J., Umhoefer H.M., Han J.Y., Lamming D.W., Davis D.B. (2017). Interleukin 6 protects pancreatic beta cells from apoptosis by stimulation of autophagy. FASEB J..

[40] Wang Y., van Boxel-Dezaire A.H., Cheon H., Yang J., Stark G.R. (2013). STAT3 activation in response to IL-6 is prolonged by the binding of IL-6 receptor to EGF receptor. Proc. Natl. Acad. Sci. USA.

[41] Hilfiker-Kleiner D., Hilfiker A., Fuchs M., Kaminski K., Schaefer A., Schieffer B., Hillmer A., Schmiedl A., Ding Z., Podewski E. (2004). Signal transducer and activator of transcription 3 is required for myocardial capillary growth, control of interstitial matrix deposition, and heart protection from ischemic injury. Circ. Res..

[42] Dawn B., Xuan Y.T., Guo Y., Rezazadeh A., Stein A.B., Hunt G., Wu W.J., Tan W., Bolli R. (2004). IL-6 plays an obligatory role in late preconditioning via JAK-STAT signaling and upregulation of iNOS and COX-2. Cardiovasc. Res..

[43] Klionsky D.J., Abdelmohsen K., Abe A., Abedin M.J., Abeliovich H., Acevedo Arozena A., Adachi H., Adams C.M., Adams P.D., Adeli K. (2016). Guidelines for the use and interpretation of assays for monitoring autophagy (3rd edition). Autophagy.

[44] Rubinsztein D.C., Cuervo A.M., Ravikumar B., Sarkar S., Korolchuk V., Kaushik S., Klionsky D.J. (2009). In search of an “autophagomometer”. Autophagy.

[45] Sarkar S., Ravikumar B., Rubinsztein D.C. (2009). Autophagic clearance of aggregate-prone proteins associated with neurodegeneration. Methods Enzym..

[46] Kroemer G., Dallaporta B., Resche-Rigon M. (1998). The mitochondrial death/life regulator in apoptosis and necrosis. Annu. Rev. Physiol..

[47] Crompton M. (1999). The mitochondrial permeability transition pore and its role in cell death. Biochem. J..

[48] Kroemer G., Reed J.C. (2000). Mitochondrial control of cell death. Nat. Med..

[49] Kramer D.K., Bouzakri K., Holmqvist O., Al-Khalili L., Krook A. (2005). Effect of serum replacement with plysate on cell growth and metabolismin primary cultures of human skeletal muscle. Cytotechnology.

[50] Mannello F., Tonti G.A. (2007). Concise review: No breakthroughs for human mesenchymal and embryonic stem cell culture: Conditioned medium, feeder layer, or feeder-free; medium with fetal calf serum, human serum, or enriched plasma; serum-free, serum replacement nonconditioned medium, or ad hoc formula? All that glitters is not gold!. Stem Cells.

[51] Van der Valk J., Brunner D., De Smet K., Fex Svenningsen A., Honegger P., Knudsen L.E., Lindl T., Noraberg J., Price A., Scarino M.L. (2010). Optimization of chemically defined cell culture media--replacing fetal bovine serum in mammalian in vitro methods. Toxicol. Vitr..

[52] Zheng X., Baker H., Hancock W.S., Fawaz F., McCaman M., Pungor E. (2006). Proteomic analysis for the assessment of different lots of fetal bovine serum as a raw material for cell culture. Part IV. Application of proteomics to the manufacture of biological drugs. Biotechnol. Prog..

[53] Colzani M., Waridel P., Laurent J., Faes E., Ruegg C., Quadroni M. (2009). Metabolic labeling and protein linearization technology allow the study of proteins secreted by cultured cells in serum-containing media. J. Proteome Res..

[54] Lambert K., Pirt S.J. (1979). Growth of human diploid cells (strain MRC-5) in defined medium; replacement of serum by a fraction of serum ultrafiltrate. J. Cell Sci..

[55] Codeluppi S., Gregory E.N., Kjell J., Wigerblad G., Olson L., Svensson C.I. (2011). Influence of rat substrain and growth conditions on the characteristics of primary cultures of adult rat spinal cord astrocytes. J. Neurosci. Methods.

[56] Pontarin G., Ferraro P., Rampazzo C., Kollberg G., Holme E., Reichard P., Bianchi V. (2011). Deoxyribonucleotide metabolism in cycling and resting human fibroblasts with a missense mutation in p53R2, a subunit of ribonucleotide reductase. J. Biol. Chem..

[57] Van Rechem C., Boulay G., Pinte S., Stankovic-Valentin N., Guerardel C., Leprince D. (2010). Differential regulation of HIC1 target genes by CtBP and NuRD, via an acetylation/SUMOylation switch, in quiescent versus proliferating cells. Mol. Cell Biol..

[58] Chao W., Shen Y., Zhu X., Zhao H., Novikov M., Schmidt U., Rosenzweig A. (2005). Lipopolysaccharide improves cardiomyocyte survival and function after serum deprivation. J. Biol. Chem..

[59] Borlongan C.V., Yamamoto M., Takei N., Kumazaki M., Ungsuparkorn C., Hida H., Sanberg P.R., Nishino H. (2000). Glial cell survival is enhanced during melatonin-induced neuroprotection against cerebral ischemia. FASEB J..

[60] Chen Y., Wang H., Zhang Y., Wang Z., Liu S., Cui L. (2019). Pretreatment of ghrelin protects H9c2 cells against hypoxia/reoxygenation-induced cell death via PI3K/AKT and AMPK pathways. Artif. Cells Nanomed. Biotechnol..

[61] Zhao J., Jie Q., Li G., Li Y., Liu B., Li H., Luo J., Qin X., Li Z., Wei Y. (2018). Rac1 promotes the survival of H9c2 cells during serum deficiency targeting JNK/c-JUN/Cyclin-D1 and AKT2/MCL1 pathways. Int. J. Med. Sci..

[62] Yellon D.M., Hausenloy D.J. (2007). Myocardial reperfusion injury. N. Engl. J. Med..

[63] Rochitte C.E., Lima J.A., Bluemke D.A., Reeder S.B., McVeigh E.R., Furuta T., Becker L.C., Melin J.A. (1998). Magnitude and time course of microvascular obstruction and tissue injury after acute myocardial infarction. Circulation.

[64] Zhao Z.Q., Nakamura M., Wang N.P., Velez D.A., Hewan-Lowe K.O., Guyton R.A., Vinten-Johansen J. (2000). Dynamic progression of contractile and endothelial dysfunction and infarct extension in the late phase of reperfusion. J. Surg Res..

[65] Qin Z.H., Wang Y., Kegel K.B., Kazantsev A., Apostol B.L., Thompson L.M., Yoder J., Aronin N., DiFiglia M. (2003). Autophagy regulates the processing of amino terminal huntingtin fragments. Hum. Mol. Genet..

[66] Shintani T., Klionsky D.J. (2004). Autophagy in health and disease: A double-edged sword. Science.

[67] Matsui Y., Kyoi S., Takagi H., Hsu C.P., Hariharan N., Ago T., Vatner S.F., Sadoshima J. (2008). Molecular mechanisms and physiological significance of autophagy during myocardial ischemia and reperfusion. Autophagy.

[68] Sala-Mercado J.A., Wider J., Undyala V.V., Jahania S., Yoo W., Mentzer R.M., Gottlieb R.A., Przyklenk K. (2010). Profound cardioprotection with chloramphenicol succinate in the swine model of myocardial ischemia-reperfusion injury. Circulation.

[69] Yan L., Vatner D.E., Kim S.J., Ge H., Masurekar M., Massover W.H., Yang G., Matsui Y., Sadoshima J., Vatner S.F. (2005). Autophagy in chronically ischemic myocardium. Proc. Natl. Acad. Sci. USA.

[70] Gedik N., Thielmann M., Kottenberg E., Peters J., Jakob H., Heusch G., Kleinbongard P. (2014). No evidence for activated autophagy in left ventricular myocardium at early reperfusion with protection by remote ischemic preconditioning in patients undergoing coronary artery bypass grafting. PLoS ONE.

[71] Zhu J., Lu T., Yue S., Shen X., Gao F., Busuttil R.W., Kupiec-Weglinski J.W., Xia Q., Zhai Y. (2015). Rapamycin protection of livers from ischemia and reperfusion injury is dependent on both autophagy induction and mammalian target of rapamycin complex 2-Akt activation. Transplantation.

[72] Zhang Y.L., Zhang J., Cui L.Y., Yang S. (2015). Autophagy activation attenuates renal ischemia-reperfusion injury in rats. Exp. Biol. Med..

[73] Abdul-Ghani S., Heesom K.J., Angelini G.D., Suleiman M.S. (2014). Cardiac phosphoproteomics during remote ischemic preconditioning: A role for the sarcomeric Z-disk proteins. Biomed. Res. Int..

[74] Gedik N., Kottenberg E., Thielmann M., Frey U.H., Jakob H., Peters J., Heusch G., Kleinbongard P. (2017). Potential humoral mediators of remote ischemic preconditioning in patients undergoing surgical coronary revascularization. Sci. Rep..

[75] Szczepanek K., Chen Q., Derecka M., Salloum F.N., Zhang Q., Szelag M., Cichy J., Kukreja R.C., Dulak J., Lesnefsky E.J. (2011). Mitochondrial-targeted Signal transducer and activator of transcription 3 (STAT3) protects against ischemia-induced changes in the electron transport chain and the generation of reactive oxygen species. J. Biol. Chem..

[76] Yeh C.C., Li H., Malhotra D., Turcato S., Nicholas S., Tu R., Zhu B.Q., Cha J., Swigart P.M., Myagmar B.E. (2010). Distinctive ERK and p38 signaling in remote and infarcted myocardium during post-MI remodeling in the mouse. J. Cell Biochem..

[77] Yin T., Sandhu G., Wolfgang C.D., Burrier A., Webb R.L., Rigel D.F., Hai T., Whelan J. (1997). Tissue-specific pattern of stress kinase activation in ischemic/reperfused heart and kidney. J. Biol. Chem..

[78] Li J., Xuan W., Yan R., Tropak M.B., Jean-St-Michel E., Liang W., Gladstone R., Backx P.H., Kharbanda R.K., Redington A.N. (2011). Remote preconditioning provides potent cardioprotection via PI3K/Akt activation and is associated with nuclear accumulation of beta-catenin. Clin. Sci..

[79] Kim H.S., Loughran P.A., Billiar T.R. (2008). Carbon monoxide decreases the level of iNOS protein and active dimer in IL-1beta-stimulated hepatocytes. Nitric. Oxide..

[80] Zhai P., Sadoshima J. (2012). Glycogen synthase kinase-3beta controls autophagy during myocardial ischemia and reperfusion. Autophagy.

[81] Zhai P., Sciarretta S., Galeotti J., Volpe M., Sadoshima J. (2011). Differential roles of GSK-3beta during myocardial ischemia and ischemia/reperfusion. Circ. Res..

[82] Hu Z., Hu S., Yang S., Chen M., Zhang P., Liu J., Abbott G.W. (2016). Remote Liver Ischemic Preconditioning Protects against Sudden Cardiac Death via an ERK/GSK-3beta-Dependent Mechanism. PLoS ONE.

[83] Runwal G., Stamatakou E., Siddiqi F.H., Puri C., Zhu Y., Rubinsztein D.C. (2019). LC3-positive structures are prominent in autophagy-deficient cells. Sci. Rep..

[84] Bjorkoy G., Lamark T., Pankiv S., Overvatn A., Brech A., Johansen T. (2009). Monitoring autophagic degradation of p62/SQSTM1. Methods Enzym..

[85] Heusch G. (2017). Remote Ischemic Conditioning in Cardiovascular Surgery. J. Cardiovasc. Pharmacol..

[86] Zangrillo A., Musu M., Greco T., Di Prima A.L., Matteazzi A., Testa V., Nardelli P., Febres D., Monaco F., Calabro M.G. (2015). Additive Effect on Survival of Anaesthetic Cardiac Protection and Remote Ischemic Preconditioning in Cardiac Surgery: A Bayesian Network Meta-Analysis of Randomized Trials. PLoS ONE.

[87] Zhou C., Liu Y., Yao Y., Zhou S., Fang N., Wang W., Li L. (2013). beta-blockers and volatile anesthetics may attenuate cardioprotection by remote preconditioning in adult cardiac surgery: A meta-analysis of 15 randomized trials. J. Cardiothorac. Vasc. Anesth..

[88] Noh H.S., Shin I.W., Ha J.H., Hah Y.S., Baek S.M., Kim D.R. (2010). Propofol protects the autophagic cell death induced by the ischemia/reperfusion injury in rats. Mol. Cells.

[89] Qiao S., Xie H., Wang C., Wu X., Liu H., Liu C. (2013). Delayed anesthetic preconditioning protects against myocardial infarction via activation of nuclear factor-kappaB and upregulation of autophagy. J. Anesth..

[90] Kersten J.R., Schmeling T.J., Pagel P.S., Gross G.J., Warltier D.C. (1997). Isoflurane mimics ischemic preconditioning via activation of K (ATP) channels: Reduction of myocardial infarct size with an acute memory phase. Anesthesiology.

[91] Behmenburg F., van Caster P., Bunte S., Brandenburger T., Heinen A., Hollmann M.W., Huhn R. (2018). Impact of Anesthetic Regimen on Remote Ischemic Preconditioning in the Rat Heart in vivo. Anesth. Analg..

[92] Bunte S., Behmenburg F., Eckelskemper F., Mohr F., Stroethoff M., Raupach A., Heinen A., Hollmann M.W., Huhn R. (2019). Cardioprotection by Humoral Factors Released After Remote Ischemic Preconditioning Depends on Anesthetic Regimen. Crit. Care Med..

[93] Berger M.M., Huhn R., Oei G.T., Heinen A., Winzer A., Bauer I., Preckel B., Weber N.C., Schlack W., Hollmann M.W. (2010). Hypoxia induces late preconditioning in the rat heart in vivo. Anesthesiology.

[94] Sheng R., Zhang T.T., Felice V.D., Qin T., Qin Z.H., Smith C.D., Sapp E., Difiglia M., Waeber C. (2014). Preconditioning stimuli induce autophagy via sphingosine kinase 2 in mouse cortical neurons. J. Biol. Chem..

[95] Heusch G., Musiolik J., Gedik N., Skyschally A. (2011). Mitochondrial STAT3 activation and cardioprotection by ischemic postconditioning in pigs with regional myocardial ischemia/reperfusion. Circ. Res..

[96] Heusch G., Musiolik J., Kottenberg E., Peters J., Jakob H., Thielmann M. (2012). STAT5 activation and cardioprotection by remote ischemic preconditioning in humans: Short communication. Circ. Res..

[97] Pepe S., Liaw N.Y., Hepponstall M., Sheeran F.L., Yong M.S., d’Udekem Y., Cheung M.M., Konstantinov I.E. (2013). Effect of remote ischemic preconditioning on phosphorylated protein signaling in children undergoing tetralogy of Fallot repair: A randomized controlled trial. J. Am. Heart Assoc..

[98] Wu Q., Wang T., Chen S., Zhou Q., Li H., Hu N., Feng Y., Dong N., Yao S., Xia Z. (2018). Cardiac protective effects of remote ischaemic preconditioning in children undergoing tetralogy of fallot repair surgery: A randomized controlled trial. Eur. Heart J..

[99] Liang Y., Li Y.P., He F., Liu X.Q., Zhang J.Y. (2015). Long-term, regular remote ischemic preconditioning improves endothelial function in patients with coronary heart disease. Braz. J. Med. Biol Res..

[100] Hausenloy D.J., Yellon D.M. (2013). Myocardial ischemia-reperfusion injury: A neglected therapeutic target. J. Clin. Investig..

[101] Shimizu M., Konstantinov I.E., Kharbanda R.K., Cheung M.H., Redington A.N. (2007). Effects of intermittent lower limb ischaemia on coronary blood flow and coronary resistance in pigs. Acta Physiol..

[102] Kono Y., Fukuda S., Hanatani A., Nakanishi K., Otsuka K., Taguchi H., Shimada K. (2014). Remote ischemic conditioning improves coronary microcirculation in healthy subjects and patients with heart failure. Drug Des. Dev..

[103] Iwai-Kanai E., Yuan H., Huang C., Sayen M.R., Perry-Garza C.N., Kim L., Gottlieb R.A. (2008). A method to measure cardiac autophagic flux in vivo. Autophagy.

[104] Zhu H., Tannous P., Johnstone J.L., Kong Y., Shelton J.M., Richardson J.A., Le V., Levine B., Rothermel B.A., Hill J.A. (2007). Cardiac autophagy is a maladaptive response to hemodynamic stress. J. Clin. Investig..

[105] Abas L., Bogoyevitch M.A., Guppy M. (2000). Mitochondrial ATP production is necessary for activation of the extracellular-signal-regulated kinases during ischaemia/reperfusion in rat myocyte-derived H9c2 cells. Biochem. J..

[106] Kuznetsov A.V., Javadov S., Sickinger S., Frotschnig S., Grimm M. (2015). H9c2 and HL-1 cells demonstrate distinct features of energy metabolism, mitochondrial function and sensitivity to hypoxia-reoxygenation. Biochim. Biophys. Acta.

[107] Kimes B.W., Brandt B.L. (1976). Properties of a clonal muscle cell line from rat heart. Exp. Cell Res..

[108] Mejia-Alvarez R., Tomaselli G.F., Marban E. (1994). Simultaneous expression of cardiac and skeletal muscle isoforms of the L-type Ca2+ channel in a rat heart muscle cell line. J. Physiol..

[109] Botker H.E., Hausenloy D., Andreadou I., Antonucci S., Boengler K., Davidson S.M., Deshwal S., Devaux Y., Di Lisa F., Di Sante M. (2018). Practical guidelines for rigor and reproducibility in preclinical and clinical studies on cardioprotection. Basic Res. Cardiol..

[110] Zheng Q., Wang X. (2010). Autophagy and the ubiquitin-proteasome system in cardiac dysfunction. Panminerva Med..

[111] Wang X., Robbins J. (2006). Heart failure and protein quality control. Circ. Res..

[112] Hu C., Tian Y., Xu H., Pan B., Terpstra E.M., Wu P., Wang H., Li F., Liu J., Wang X. (2018). Inadequate ubiquitination-proteasome coupling contributes to myocardial ischemia-reperfusion injury. J. Clin. Investig..

[113] Divald A., Kivity S., Wang P., Hochhauser E., Roberts B., Teichberg S., Gomes A.V., Powell S.R. (2010). Myocardial ischemic preconditioning preserves postischemic function of the 26S proteasome through diminished oxidative damage to 19S regulatory particle subunits. Circ. Res..

[114] Billah M., Ridiandries A., Rayner B.S., Allahwala U.K., Dona A., Khachigian L.M., Bhindi R. (2019). Egr-1 functions as a master switch regulator of remote ischemic preconditioning-induced cardioprotection. Basic Res. Cardiol..

[115] Li C., Jackson R.M. (2002). Reactive species mechanisms of cellular hypoxia-reoxygenation injury. Am. J. Physiol. Cell Physiol..

[116] Webster K.A., Discher D.J., Kaiser S., Hernandez O., Sato B., Bishopric N.H. (1999). Hypoxia-activated apoptosis of cardiac myocytes requires reoxygenation or a pH shift and is independent of p53. J. Clin. Investig..

[117] Shang L., Ananthakrishnan R., Li Q., Quadri N., Abdillahi M., Zhu Z., Qu W., Rosario R., Toure F., Yan S.F. (2010). RAGE modulates hypoxia/reoxygenation injury in adult murine cardiomyocytes via JNK and GSK-3beta signaling pathways. PLoS ONE.

[118] Calvillo L., Vanoli E., Andreoli E., Besana A., Omodeo E., Gnecchi M., Zerbi P., Vago G., Busca G., Schwartz P.J. (2011). Vagal stimulation, through its nicotinic action, limits infarct size and the inflammatory response to myocardial ischemia and reperfusion. J. Cardiovasc. Pharmacol..

[119] Guo J., Zhu J., Ma L., Shi H., Hu J., Zhang S., Hou L., Xu F., An Y., Yu H. (2018). Chronic Kidney Disease Exacerbates Myocardial Ischemia Reperfusion Injury: Role of Endoplasmic Reticulum Stress-Mediated Apoptosis. Shock.

[120] Bohl S., Medway D.J., Schulz-Menger J., Schneider J.E., Neubauer S., Lygate C.A. (2009). Refined approach for quantification of in vivo ischemia-reperfusion injury in the mouse heart. Am. J. Physiol. Heart Circ. Physiol..

[121] Deng C., Sun Z., Tong G., Yi W., Ma L., Zhao B., Cheng L., Zhang J., Cao F., Yi D. (2013). alpha-Lipoic acid reduces infarct size and preserves cardiac function in rat myocardial ischemia/reperfusion injury through activation of PI3K/Akt/Nrf2 pathway. PLoS ONE.

[122] Price A.N., Cheung K.K., Lim S.Y., Yellon D.M., Hausenloy D.J., Lythgoe M.F. (2011). Rapid assessment of myocardial infarct size in rodents using multi-slice inversion recovery late gadolinium enhancement CMR at 9.4T. J. Cardiovasc. Magn. Reson..

[123] Li X., Jia P., Huang Z., Liu S., Miao J., Guo Y., Wu N., Jia D. (2019). Lycopene protects against myocardial ischemia-reperfusion injury by inhibiting mitochondrial permeability transition pore opening. Drug Des. Dev..

[124] Sun D., Huang J., Zhang Z., Gao H., Li J., Shen M., Cao F., Wang H. (2012). Luteolin limits infarct size and improves cardiac function after myocardium ischemia/reperfusion injury in diabetic rats. PLoS ONE.

[125] Tsuda T., Gao E., Evangelisti L., Markova D., Ma X., Chu M.L. (2003). Post-ischemic myocardial fibrosis occurs independent of hemodynamic changes. Cardiovasc. Res..

[126] Eckle T., Grenz A., Kohler D., Redel A., Falk M., Rolauffs B., Osswald H., Kehl F., Eltzschig H.K. (2006). Systematic evaluation of a novel model for cardiac ischemic preconditioning in mice. Am. J. Physiol. Heart Circ. Physiol..

[127] Tanase H., Yamori Y., Hansen C.T., Lovenberg W. (1982). Heart size in inbred strains of rats. Part 1. Genetic determination of the development of cardiovascular enlargement in rats. Hypertension.

[128] Cao C.M., Zhang Y., Weisleder N., Ferrante C., Wang X., Lv F., Zhang Y., Song R., Hwang M., Jin L. (2010). MG53 constitutes a primary determinant of cardiac ischemic preconditioning. Circulation.

[129] Shen Y.T., Depre C., Yan L., Park J.Y., Tian B., Jain K., Chen L., Zhang Y., Kudej R.K., Zhao X. (2008). Repetitive ischemia by coronary stenosis induces a novel window of ischemic preconditioning. Circulation.

[130] Kabeya Y., Mizushima N., Ueno T., Yamamoto A., Kirisako T., Noda T., Kominami E., Ohsumi Y., Yoshimori T. (2000). LC3, a mammalian homologue of yeast Apg8p, is localized in autophagosome membranes after processing. EMBO J..

[131] Goldman A., Harper S., Speicher D.W. (2016). Detection of Proteins on Blot Membranes. Curr. Protoc. Protein Sci..

[132] Tremblay F., Brule S., Hee Um S., Li Y., Masuda K., Roden M., Sun X.J., Krebs M., Polakiewicz R.D., Thomas G. (2007). Identification of IRS-1 Ser-1101 as a target of S6K1 in nutrient- and obesity-induced insulin resistance. Proc. Natl. Acad. Sci. USA.

[133] Yung H.W., Charnock-Jones D.S., Burton G.J. (2011). Regulation of AKT phosphorylation at Ser473 and Thr308 by endoplasmic reticulum stress modulates substrate specificity in a severity dependent manner. PLoS ONE.

[134] Aldridge G.M., Podrebarac D.M., Greenough W.T., Weiler I.J. (2008). The use of total protein stains as loading controls: An alternative to high-abundance single-protein controls in semi-quantitative immunoblotting. J. Neurosci. Methods.

[135] Eaton S.L., Roche S.L., Llavero Hurtado M., Oldknow K.J., Farquharson C., Gillingwater T.H., Wishart T.M. (2013). Total protein analysis as a reliable loading control for quantitative fluorescent Western blotting. PLoS ONE.

