# Correction: Billah et al. Remote Ischemic Preconditioning Induces Cardioprotective Autophagy and Signals Through the IL-6-Dependent JAK-STAT Pathway. *Int. J. Mol. Sci.* 2020, *21*, 1692

**DOI:** 10.3390/ijms27156866

**Published:** 2026-07-31

**Authors:** Muntasir Billah, Anisyah Ridiandries, Usaid K Allahwala, Harshini Mudaliar, Anthony Dona, Stephen Hunyor, Levon M. Khachigian, Ravinay Bhindi

**Affiliations:** 1 Department of Cardiology, Kolling Institute of Medical Research, Northern Sydney Local Health District, St Leonards, NSW 2065, Australia; 2 Sydney Medical School Northern, University of Sydney, Sydney, NSW 2006, Australia; 3 School of Life Sciences, Independent University Bangladesh, Dhaka 1229, Bangladesh; 4 Vascular Biology and Translational Research, School of Medical Sciences, University of New South Wales, Sydney, NSW 2052, Australia

In the original publication [1], there was a mistake in Figure 1 as published. There was an inadvertent assembly error in the LC3 blot in Figure 1B and the Figure 1 legend was incomplete. The corrected Figure 1 and its legend appear below. The authors state that the scientific conclusions are unaffected. This correction was approved by the Academic Editor. The original publication has also been updated.

## Figures and Tables

**Figure 1:**
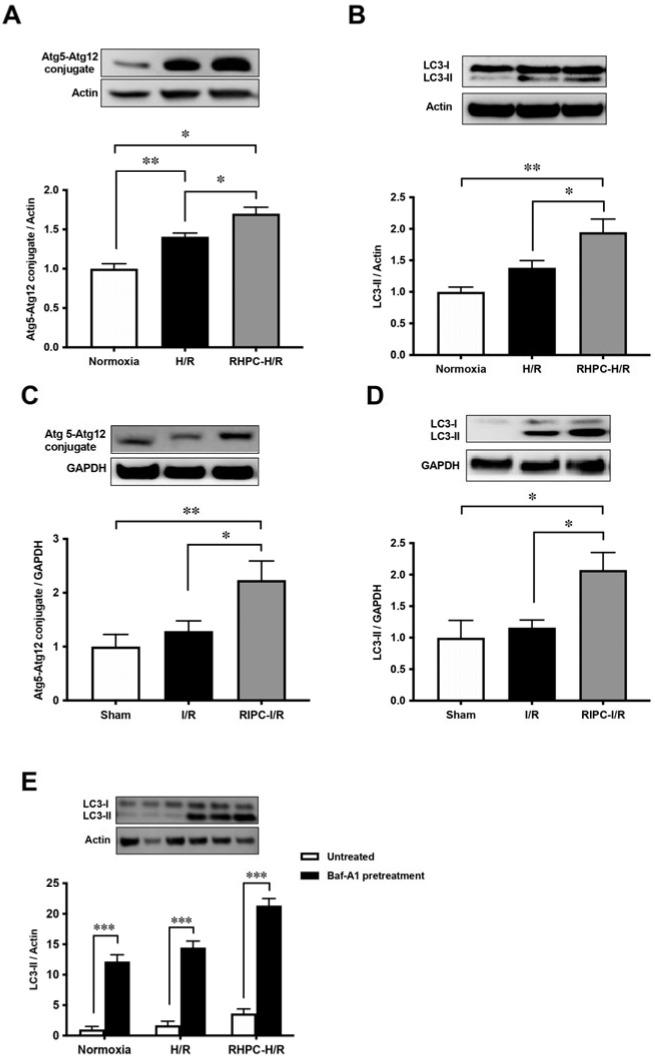
Effect of RIPC prior to I/R on autophagy protein expression in vitro and in vivo. Western blot analysis and representative images of (**A**) Atg5-Atg12 conjugate levels in H9c2 cells, (**B**) LC3 protein levels in H9c2 cells, (**C**) Atg5-Atg12 conjugate levels in rat heart lysate, (**D**) LC3 protein levels in rat heart lysate, and (**E**) LC3 protein levels in bafilomycin-A1-treated H9c2 cells, expressed as mean ± SEM, fold relative to control; *n* = at least 3 independent experiments, * *p* < 0.05, ** *p* < 0.01, *** *p* < 0.001.
