# Supplementary material for: Remote Ischemic Preconditioning Induces Cardioprotective Autophagy and Signals through the IL-6-Dependent JAK-STAT Pathway
Source: Int J Mol Sci. 2020 Mar 1;21(5):1692. doi: 10.3390/ijms21051692 (PMC7084188; doi:10.3390/ijms21051692)
Supplement: Supplementary file 1 [file ijms-21-01692-s001.pdf]

Supplementary Materials for Billah et al.

Remote Ischemic preconditioning induces cardioprotective autophagy and signals through the IL-6-dependent JAK-STAT pathway

Blood collection

Immediately post-RIPC, abdominal incision was made on the anesthetized rats and renal artery visualized, and 3–4 mL of blood was withdrawn with a 22-gauge hypodermic needle and quickly transferred into a Lithium-Heparin Blood collection tube (BD Biosciences, New Jersey, USA) and kept on ice till centrifugation. Blood was centrifuged at 2500 rpm for 15 min. Blood plasma was collected in a 1.5-mL Eppendorf tube and stored at −80°C.

Flow cytometry

1. In vitro apoptosis study

After the experiments, H9c2 cells were washed with cold D-PBS twice. Followed by trypsinization, cells were centrifuged at 1000 rpm for 5 min. Cell pellet was suspended in 1x binding buffer at a concentration of 1 x 10^6 cells/mL. In total, 100µL of the solution was transferred to FACS tubes. Then, 5µL of Annexin V FITC and 5µL of propidium iodide (PI) was added to the tube and incubated for 15 min at room temperature in the dark. Prior to running the sample in the flow cytometry machine, 400µL of 1x binding buffer was added to each tube and the apoptotic cells were determined by analyzing 10,000 gated cells using a BD FACS flow cytometer.

2. Mitochondrial membrane potential loss study

After the experiments, H9c2 cells were washed with cold D-PBS twice. Followed by trypsinization, cells were centrifuged at 1000 rpm for 5 min. Cell pellet was suspended in 1mL of D-PBS at a concentration of 1 x 10^6 cells/mL. Briefly, 5µL of 2µM stock of DiLC5 dye was added to the cells and incubated at room temperature for 15 min in the dark. The fluorescence intensity of the cells was analyzed by gating 10,000 cells using a BD FACS flow cytometer.

Cell viability assay

Cell viability was measured using a Live/Dead Cell Imaging Kit (#R37601, Thermofisher Scientific, Massachusetts, USA). Cells were seeded in a 6-well plate, and after experiments, wells were washed 2 times with D-PBS. Live Green vial from the kit was thawed and transferred to the Dead Red vial to prepare 2x stock. Equal volumes of 2x stock and live cell imaging solution (140mM NaCl, 2.5mM KCl, 1.8 mM $CaCl_2$, 1.0 mM $MgCl_2$, 20mM HEPES, pH 7.4) were incubated at room temperature for 15 min. Fluorescence images were taken using GFP and Texas Red filter in an EVOS FL Auto Cell Imaging System (Thermofisher Scientific, Massachusetts, USA).

Real-Time Polymerase Chain Reaction assay

1. Preparation of sample for RNA extraction

After exposure to the experimental condition, cells were washed with ice-cold D-PBS, and trypsinized cells ($5x10^6$ to $1x10^7$ cells) were centrifuged at 14,000 rpm. Cell pellet was resuspended in 500µL of lysis solution/ 2-ME mixture, and total RNA extracted using the Gen elute Mammalian Total RNA Mini Prep Kit (Sigma-Aldrich, St Louis, Missouri, USA).

2. RNA extraction from tissue sample

Tissue sample was excised and washed with ice-cold D-PBS and "snap-frozen" in dry ice. In total, 500μL of lysis solution/2-ME mixture was added to the frozen tissue and homogenized in a flat bottom DNAase, RNAse-free cell culture tube on ice with TissueRuptor (Qiagen, USA). Total RNA was isolated using the Gen elute Mammalian Total RNA Mini Prep kit.

3. Total RNA concentration measurement

The concentration of total RNA was measured from 2μL of the extracted RNA sample using a NanoDrop 1000 Spectrophotometer (Thermofisher Scientific, USA).

4. cDNA synthesis

RNA was reverse transcribed to 1μg cDNA using the Tetro cDNA Synthesis Kit (Bioline, London, UK) in accordance with the manufacturer's instructions. cDNA was stored in −20°C.

5. Real-Time PCR

RT-PCR mixture prepared using the SensiFast SYBR HI ROX Kit (Bioline, London, UK) in accordance with the manufacturer's instructions. Initially, serial dilution of cDNA sample was done, and RT-PCR was run to generate a standard curve to estimate the efficiency of the primers. RT-PCR was performed in an ABI Prism 7900 HT Sequence Detection System (Applied Biosystems, Foster City, CA). Reactions were performed in at least triplicate and were analyzed by relative quantitation using RQ Manager software, version 1.2 (Applied Biosystems).

ELISA

Enzyme-linked immunosorbent assay (ELISA) kits were used to determine the concentration of IL-6 (Rat IL-6 Quantikine ELISA Kit, R&D Systems, Minneapolis, USA) protein. For circulating protein levels, blood from rats was collected in a Lithium-Heparin tube and spun at 2500 rpm for 5 min and plasma was collected. For the detection of plasma IL-6, 50 μL of rat plasma was used and protein estimated as per the manufacturer's instructions.

Autophagy promotion and inhibition

Recombinant IL-6 was added to hypoxic media, and H9c2 cells were exposed to recombinant IL-6-containing hypoxic media during 30 min of hypoxia. In order to optimize the rapamycin concentration to induce autophagy, we treated overnight serum-starved H9c2 cells with different concentrations of rapamycin for 4 h in serum-free DMEM. 3-MA blocks autophagic sequestration and autophagosome formation by inhibiting class III phosphatidylinositol 3-kinase (PI3K) [1,2]. Thus, 3-MA downregulates autophagic activity at the early stage and is widely used to understand the role of autophagy [3–5]. In previous studies, pre-treatment with 10 mM 3-MA for 2 h has been shown to effectively block autophagic activity [6]. Hence, H9c2 cells were treated with 10nM 3-MA for 2 h in serum-free DMEM. Cells were pre-treated with 200 μM Tyrphostin AG 490 for 1 h prior to exposing the cells to H/R to inhibit the JAK-STAT pathway as previously described [7].

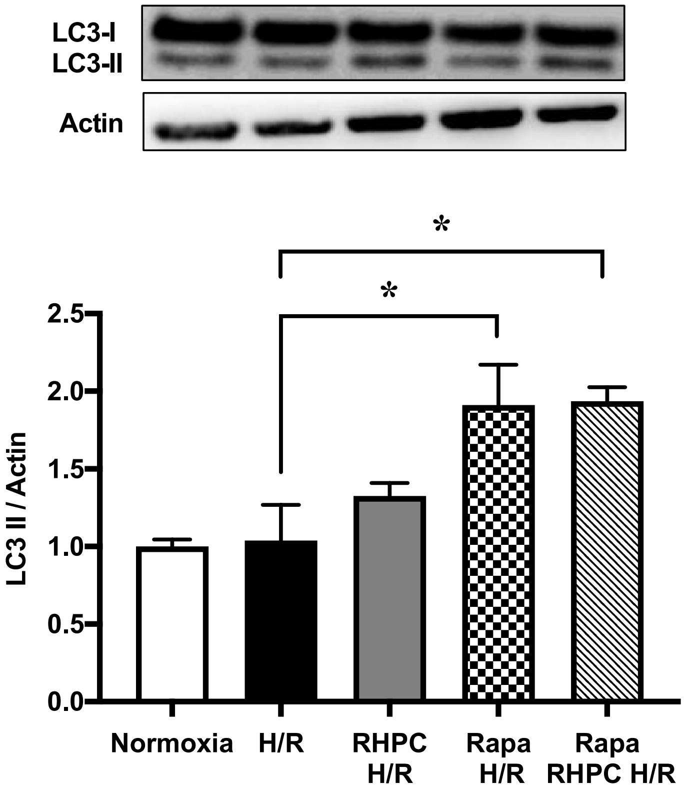

Supplementary Figure 1. Rapamycin treatment prior to H/R increases autophagic activity. Representative Western blot of LC3 protein levels in H9c2 cells treated with 1μM rapamycin for 4 h prior to exposing them to H/R and RHPC-H/R, expressed as mean ± SEM, *n*= at least 3 independent experiments; fold relative to normoxic control; **p* < 0.05.

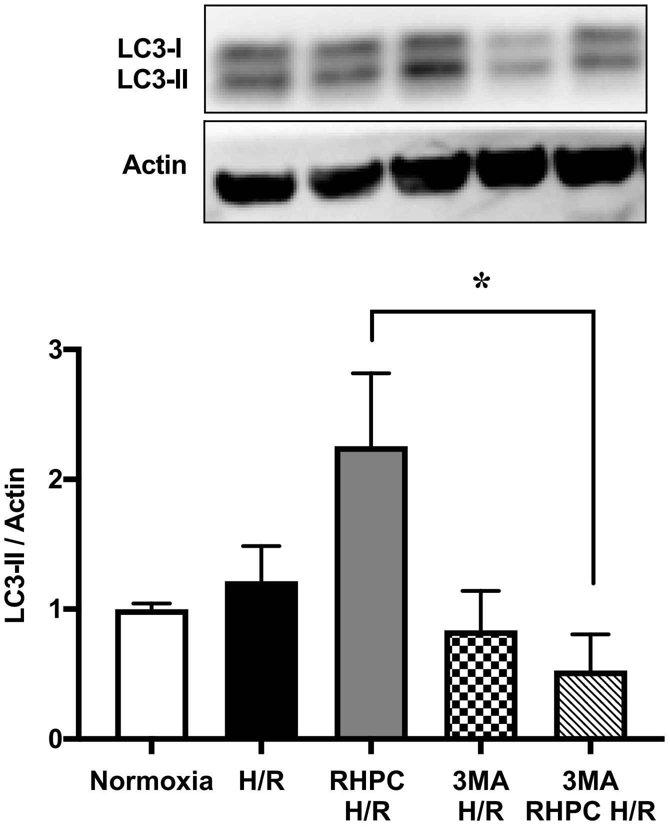

Supplementary Figure 2. Autophagy level after 3-MA treatment. H9c2 cells subjected to 3-MA treatment prior to exposures to H/R and RHPC-H/R. Western blot analysis of LC3 protein levels expressed as mean ± SEM, fold relative to the normoxic control; *n*= at least 3 independent experiments, *$p < 0.05$.

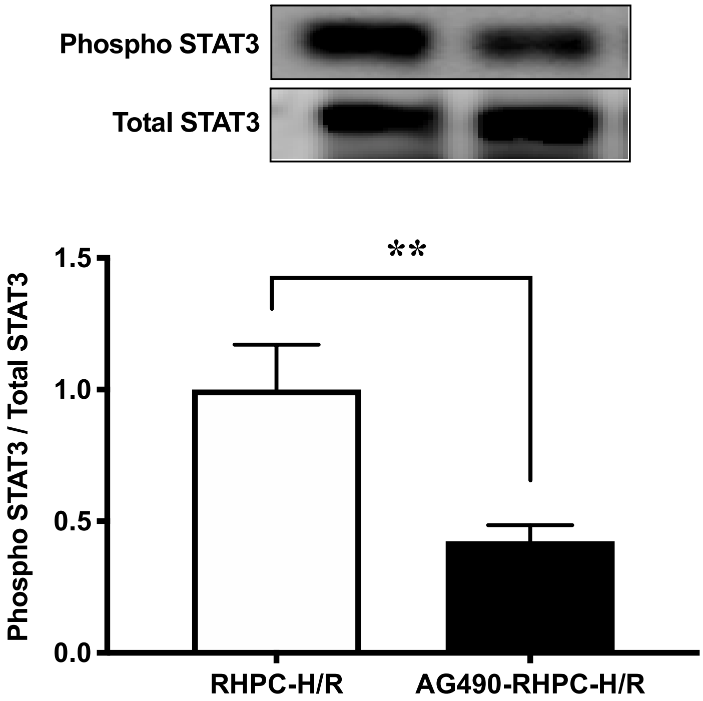

Supplementary Figure 3. Effect of tyrphostin AG-490 on STAT3 phosphorylation. H9c2 cells exposed to RHPC-H/R with and without JAK-STAT pathway inhibitor tyrphostin AG-490 pre-treatment. Representative immunoblots and statistical data of phosphorylated STAT3 are expressed as mean ± SEM, fold relative to control; *n*= at least 3 independent experiments, ***p* < 0.01.

References

1. Seglen, P.O.; Gordon, P.B., 3-Methyladenine: Specific inhibitor of autophagic/lysosomal protein degradation in isolated rat hepatocytes. *Proc. Nat. Acad Sci. USA* **1982,** *79*, 1889–1892.
2. Klionsky, D.J.; Cuervo, A.M.; Seglen, P.O., Methods for monitoring autophagy from yeast to human. *Autophagy* **2007,** *3,* 181-206.
3. Suzuki, C.; Isaka, Y.; Takabatake, Y.; Tanaka, H.; Koike, M.; Shibata, M.; Uchiyama, Y.; Takahara, S.; Imai, E., Participation of autophagy in renal ischemia/reperfusion injury. *Biochem. Biophys. Res. Commun.* **2008,** *368*, 100–106.
4. Jiang, M.; Liu, K.; Luo, J.; Dong, Z., Autophagy is a renoprotective mechanism during in vitro hypoxia and in vivo ischemia-reperfusion injury. *Am. J. Pathol.* **2010,** *176*, 1181–1192.
5. Jin, Y.; Wang, H.; Cui, X.; Jin, Y.; Xu, Z., Role of autophagy in myocardial reperfusion injury. *Front. Biosci.* **2010**, *2*, 1147–1153.
6. Wu, X.; He, L.; Chen, F.; He, X.; Cai, Y.; Zhang, G.; Yi, Q.; He, M.; Luo, J., Impaired autophagy contributes to adverse cardiac remodeling in acute myocardial infarction. *PLoS ONE* **2014,** *9*, e112891.
7. Mudaliar, H.; Rayner, B.; Billah, M.; Kapoor, N.; Lay, W.; Dona, A.; Bhindi, R., Remote ischemic preconditioning attenuates EGR-1 expression following myocardial ischemia reperfusion injury through activation of the JAK-STAT pathway. *Int. J. Cardiol.* **2017**, *228*, 729–741.

.
